# Deciphering the regulatory mechanisms of the cAMP/protein kinase A pathway and their roles in the pathogenicity of *Candida auris*

**DOI:** 10.1128/spectrum.02152-23

**Published:** 2023-09-06

**Authors:** Ji-Seok Kim, Kyung-Tae Lee, Yong-Sun Bahn

**Affiliations:** 1 Department of Biotechnology, College of Life Science and Biotechnology, Yonsei University, Seoul, South Korea; 2 Korea Zoonosis Research Institute, Jeonbuk National University, Jeonbuk, South Korea; Universidade de Sao Paulo, Ribeirao Preto, Sao Paulo, Brazil

**Keywords:** *C. auris*, Ras1, Cdc25, Ira1, Gpr1, Gpa2, Pde1, Pde2, biofilm, glycogen, nutrient starvation, virulence

## Abstract

The emergence of multidrug-resistant fungal pathogens is a significant concern for global public health. *Candida auris* poses a considerable threat as a multidrug-resistant fungal pathogen. Our recent study revealed that the adenylyl cyclase Cyr1 and protein kinase A (PKA) pathways play distinct and redundant roles in drug resistance and pathogenicity of *C. auris*. However, the upstream and negative feedback regulatory mechanisms of *C. auris* are not yet fully understood. In this study, we discovered that the small GTPase Ras1, along with its nucleotide exchange factor Cdc25 and GTPase-activating protein Ira2, plays a major role in regulating cAMP/PKA-dependent traits, while G-protein-coupled receptor Gpr1 and heterotrimeric G-protein α subunit Gpa2 play a minor role. Pde2 plays a major role in negative feedback regulation of the cAMP/PKA pathway, while Pde1 plays a minor role. Hyperactivation of the Ras/cAMP/PKA pathway by deleting *PDE2* or *BCY1* renders *C. auris* cells thermosensitive and susceptible to nutrient deficiency, which leads to attenuated virulence. Our study demonstrates the distinct contributions of hyperactivation of the Ras/cAMP/PKA signaling pathway to *C. auris* pathogenesis and suggests potential therapeutic targets for *C. auris-*mediated candidiasis.

**IMPORTANCE:**

*Candida auris* is a major concern as a multidrug-resistant fungal pathogen. While our previous studies highlighted the crucial roles of the cAMP/protein kinase A (PKA) pathway in regulating drug resistance, stress responses, morphogenesis, ploidy change, biofilm formation, and pathogenicity in this pathogen, their regulatory mechanism remains unclear. In our study, we provided evidence that the cAMP/PKA signaling pathway in *C. auris* is primarily governed by the small GTPase RAS rather than a G-protein-coupled receptor. Additionally, we discovered that the negative feedback regulation of cAMP, controlled by phosphodiesterases, is vital for *C. auris* virulence by promoting resistance to high temperatures and nutrient deficiencies. These findings underscore the diverse pathobiological significance of the Ras/cAMP/PKA signaling pathway in *C. auris*, shedding light on potential therapeutic targets and strategies for combating this multidrug-resistant fungal pathogen.

## INTRODUCTION

The emergence of multidrug-resistant pathogenic fungi is a significant threat to global public health, with over 150 million people experiencing severe fungal infections annually, resulting in approximately 1.5 million fatalities (1). Candidiasis alone affects about 9 million people and continues to increase (2). *Candida auris*, a multidrug-resistant pathogenic fungus, has been causing candidemia in healthcare environments worldwide since 2009 (3). It displays high resistance to current antifungal drugs, particularly azoles, with over 90% of strains in the United States being resistant to fluconazole (FCZ) (4). Therefore, there is a pressing need for novel therapeutic strategies to combat this pathogenic fungus.

The clinical symptoms of invasive *C. auris* infection are similar to those caused by other invasive *Candida* species, making it difficult to distinguish between them (5). The primary treatment for candidiasis caused by *C. auris* involves using caspofungin, micafungin, and anidulafungin due to their relatively low resistance rates in the echinocandin class of antifungal drugs (6). Secondary therapy may include amphotericin B (AMB), itraconazole, and posaconazole (PCZ), depending on the patient’s condition. The mortality rate associated with *C. auris* infection is estimated to be between 30% and 60% (7). Thus, the Centers for Disease Control and Prevention (CDC) has assigned a higher threat level to *C. auris* owing to its significant antifungal drug resistance. Furthermore, the prevalence of *C. auris* co-infection in immunocompromised COVID-19 patients is increasing (8). Therefore, there is a pressing need to comprehensively understand the mechanisms underlying antifungal drug resistance and the pathogenicity of *C. auris*.

The cAMP pathway is a well-known signaling network that significantly contributes to the growth, drug resistance, and virulence of various pathogenic fungi (9). It is activated by two upstream signaling branches, depending on the fungal species. One involves a G-protein-coupled receptor (GPCR) that undergoes a conformation change in response to carbohydrates and amino acids, releasing the active α subunit of the heterotrimer G-protein complex which then activates adenylyl cyclase (10 – 12). The second branch involves small GTPase RAS, which is a membrane-bound protein that undergoes lipid modification and is positively activated by guanine nucleotide exchange factor (GEF) and negatively regulated by GTPase-activating protein (GAP) (13). Upon activation, adenylyl cyclase catalyzes the conversion of ATP to cAMP, which binds to the regulatory subunit of protein kinase A (PKA), releasing its catalytic subunits. These catalytic subunits then translocate to the nucleus and activate a cascade of downstream transcription factors (14).

To maintain cAMP homeostasis and prevent overaccumulation, cyclic nucleotide phosphodiesterases (PDEs) play a crucial role. There are two types of PDEs: Pde1 and Pde2, which convert cAMP to AMP with low and high affinity, respectively (15, 16). In most pathogenic fungi, inhibition of the Ras/cAMP/PKA pathway reduces virulence, while hyperactivation of the cAMP/PKA pathway attenuates virulence in some cases (17 – 20). Thus, balanced modulation of the cAMP/PKA pathway is crucial for fungal pathogenicity.

Our previous research showed that adenylyl cyclase (Cyr1) and PKA subunits (Bcy1 regulatory subunit and Tpk1/2 catalytic subunits) play redundant and distinct roles in various aspects of *C. auris*, including growth, stress response, drug and disinfectant resistance, pseudohyphae and biofilm formation, ploidy switch, and virulence (21). Interestingly, we discovered that Tpk1/2 in *C. auris* has both Cyr1-dependent and independent functions, distinguishing it from other pathogenic fungi. Furthermore, we discovered that hyperactivation of the cAMP/PKA pathway (by *BCY1* deletion), rather than its inactivation (by *CYR1* or *TPK1/2* deletion), reduces virulence in *C. auris* (21). However, the upstream signaling branches and negative feedback regulatory systems of the Ras/cAMP/PKA pathways in *C. auris* are still unknown.

In this study, our primary objective was to determine the upstream signaling branch that governs the cAMP/PKA pathway in *C. auris*. To achieve this, we investigated two potential upstream candidates, GPCR and RAS, which are well-known regulators of fungal adenylyl cyclases, using reverse genetic approaches. Additionally, we aimed to unravel the PDE-dependent negative feedback regulatory mechanism of the cAMP/PKA pathway and evaluate its significance in the pathobiology of this fungal pathogen. By addressing these objectives, our study provides a comprehensive understanding of the regulatory mechanisms and pathobiological functions associated with the cAMP/PKA pathway in *C. auris*. These findings promise to develop innovative therapeutic strategies targeting this pathway for treating *C. auris-*mediated candidiasis.

## RESULTS

### Ras1 is the primary upstream regulator of the cAMP/PKA pathway

To determine potential upstream signaling branches of the cAMP/PKA pathway in *C. auris*, we searched its genome database for orthologous proteins that may be involved in GPCR and RAS signaling branches. Using this approach, we identified *C. auris* proteins with the highest orthology to Gpr1, Gpa2, and Ras1 in other *Candida* species: Gpr1 (B9J08_002066), Gpa2 (B9J08_002635), and Ras1 (B9J08_003446) (Fig. S1). These *C. auris* proteins all contain conserved domains that are typical of GPCR, Gα, or Ras proteins, as determined by a conserved protein domain search using InterPro analysis (https://www.ebi.ac.uk/interpro/) (Fig. S1). To investigate the functional roles of these genes, we generated deletion mutants for *RAS1*, *GPR1*, and *GPA2* in the clade I wild-type strain (B8441) background using a split-gene disruption cassette containing the nourseothricin resistance marker (*CaNAT*) and heat shock transformation method (Fig. S2). We also generated complemented strains to verify the phenotypic traits of the deletion mutants. These complemented strains were constructed by reintegrating the wild-type allele into its native locus (*gpr1*Δ::*GPR1*) or through ectopic integration (*ras1*Δ + *RAS1*, *gpa2*Δ + *GPA2*). These complemented strains exhibited a fully restored wild-type phenotype (Fig. S2). Additionally, we constructed *ras1*Δ *gpa2*Δ double deletion mutants to investigate whether the RAS and GPCR branches activate Cyr1 independently (Fig. S2).

**Fig 1:**
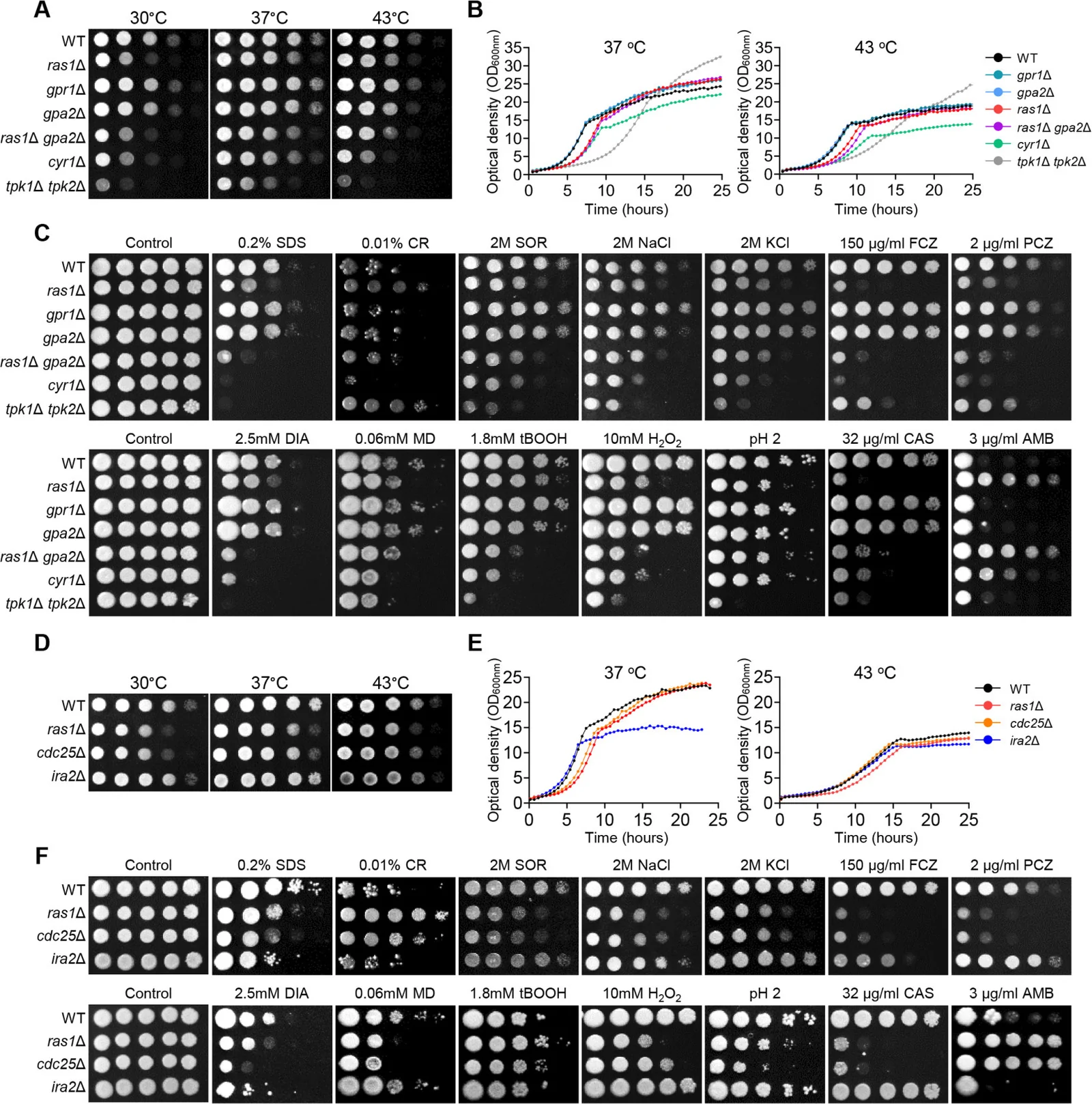
The role of the Ras1/cAMP/PKA signaling pathway in the growth and stress response of *C. auris*. (A and D) Qualitative spot assays for measuring the growth and thermotolerance of the *C. auris* wild type and mutants. (B and E) Quantitative growth rates of wild type and mutants. (C and F) Spot analysis of the *C. auris* wild type and mutants under various stress-inducing conditions, including sodium dodecyl sulfate (SDS), congo red (CR), sorbitol (SOR), sodium chloride (NaCl), potassium chloride (KCl), FCZ, PCZ, DIA, menadione (MD), *tert*-butyl hydroperoxide (tBOOH), hydrogen peroxide (H_2_O_2_), acidic pH, caspofungin (CAS), and AMB.

We conducted a comprehensive analysis of phenotypic traits in constructed mutants of Ras1, Gpr1, and Gpa2 in comparison to mutants of adenylyl cyclase (*cyr1*Δ) and PKA catalytic subunit (*tpk1*Δ *tpk2*Δ) in *C. auris*. We measured the growth rate of all mutants qualitatively and quantitatively at various temperatures (Fig. 1A and B ). The *ras1*Δ mutant showed a growth defect that was less severe than that of *cyr1*Δ, while *gpr1*Δ and *gpa2*Δ mutants displayed growth rates similar to that of the wild type at all tested temperatures. The *ras1*Δ *gpa2*Δ double mutant had a slightly more severe growth defect than every single mutant at 43°C, although not as severe as *cyr1*Δ (Fig. 1A and B). Regarding the stress response, *ras1*Δ and *cyr1*Δ exhibited similar susceptibility patterns to most stress conditions. Intriguingly, they displayed heightened susceptibility to FCZ, PCZ, and caspofungin, while exhibiting increased tolerance to AMB compared to the wild-type strain. However, they showed contrasting responses to congo red and low pH stress (Fig. 1C). These results suggest that Ras may regulate certain signaling pathways independent of Cyr1. Meanwhile, *gpr1*Δ and *gpa2*Δ were almost indistinguishable from the wild type under all tested stress conditions, while *ras1*Δ *gpa2*Δ was slightly more susceptible to diamide (DIA) and H_2_O_2_ than *ras1*Δ (Fig. 1C). Overall, our results suggest that Ras1 serves as a primary upstream regulator of Cyr1, while GPCR and other undefined upstream branches serve as secondary Cyr1 regulators in *C. auris*.

### Ras1 is positively and negatively regulated by Cdc25 and Ira2

Ras1 is regulated by GEF and GAP in *Saccharomyces cerevisiae*, where GEF (Cdc25) activates Ras1 by stimulating GDP release and GTP binding, and GAP (Ira1/2) regulates the intrinsic GTPase activity of RAS (22, 23). In *C. auris*, we found orthologs of Cdc25 and Ira1/2, with B9J08_003305 and B9J08_003924 being orthologous to Cdc25 and Ira2, respectively (Fig. S1). *Candida auris* Cdc25 contains the Ras-GEF, while Ira2 has Ras-GAP domains (Fig. S1). To investigate the functional roles of Cdc25 and Ira2, we generated *cdc25*Δ and *ira2*Δ mutants in *C. auris* (Fig. S2). Furthermore, we constructed complemented strains and verified the restoration of wild-type phenotypic characteristics (Fig. S2).

We compared the phenotypic traits of *cdc25*Δ and *ira2*Δ mutants with those of the *ras1*Δ mutant. As expected, *ras1*Δ and *cdc25*Δ mutants showed similar growth defects at varying temperatures, with similar growth patterns observed in qualitative spot assays at 30°C–43°C (Fig. 1D). The quantitative growth rate measurement showed that *ras1*Δ and *cdc25*Δ entered the stationary phase approximately 2 h later than the wild type at 37° (Fig. 1E). Additionally, *cdc25*Δ showed susceptibility to stress-inducing agents similar to that of *ras1*Δ (Fig. 1F). These results indicate that Cdc25 is the positive regulator of Ras in *C. auris*. However, *IRA2* deletion did not lead to discernable phenotypic variation, except for increased sensitivity to SDS, FCZ, and DIA, compared to the wild type (Fig. 1D through F).

### Roles of Pde1 and Pde2 for the negative feedback regulation of the cAMP/PKA pathway

**Fig 2:**
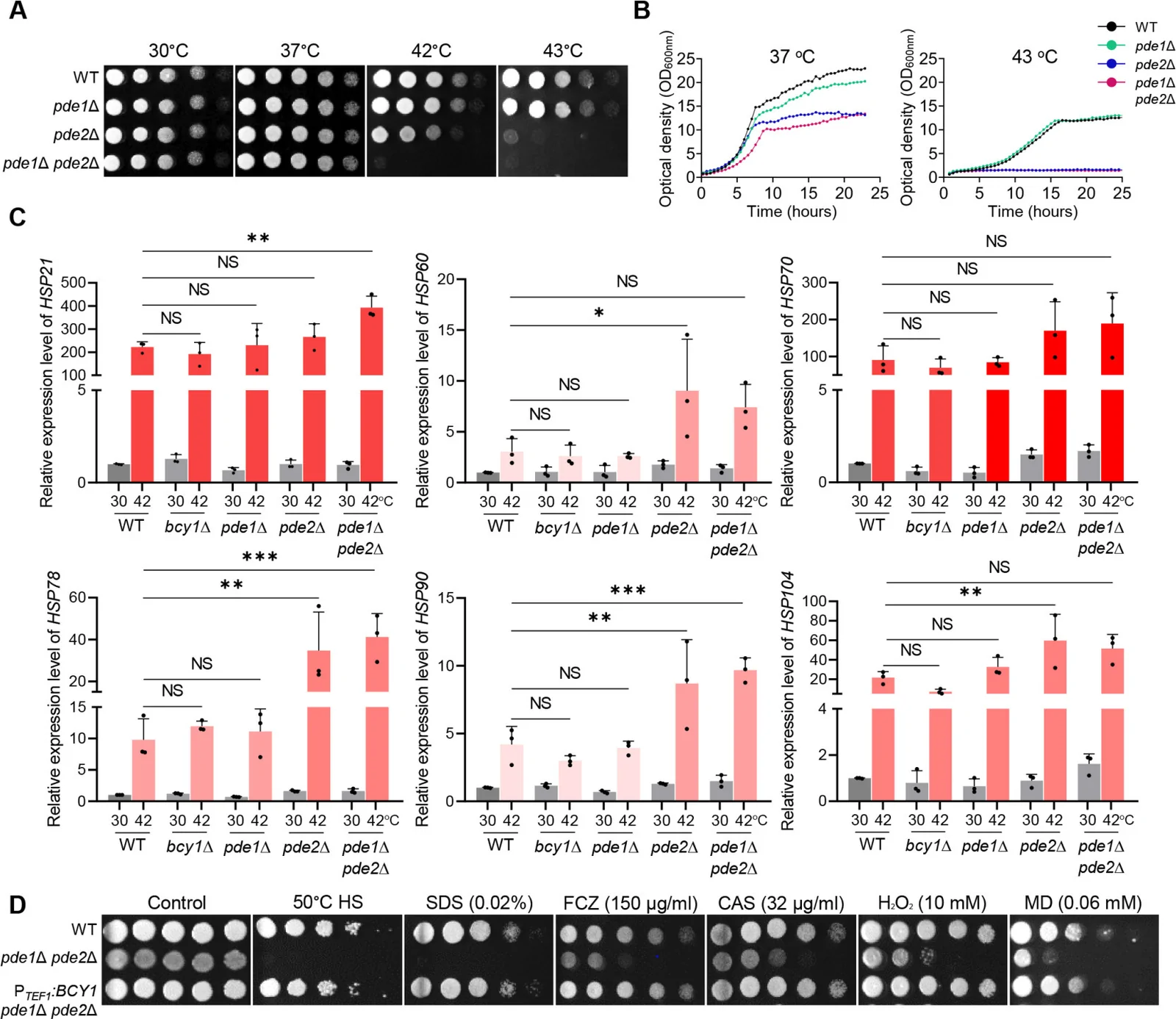
The role of PDE in thermotolerance of *C. auris*. (A) Qualitative spot assay and (B) quantitative growth rate measurement of the *C. auris* wild type and mutants. (C) Quantitative reverse transcription-PCR (RT-PCR) analysis of thermotolerance-related genes in wild type and mutants. Statistical analysis was performed using one-way analysis of variance with Bonferroni’s multiple-comparison tests (*P < 0.05, **P < 0.01, and ***P < 0.001 ). (D) Spot analysis of the wild type and mutants under various stress-inducing conditions.

The cAMP/PKA pathway is negatively regulated by low-affinity and high-affinity PDEs, Pde1 and Pde2, which can degrade cAMP to AMP (15). Lack of PDEs can cause cell toxicity due to hyperaccumulation of intracellular cAMP (24 – 28). In the *C. auris* genome database, we found Pde1 (B9J08_002549) and Pde2 (B9J08_003265) orthologs (Fig. S1). To investigate the roles of Pde1 and Pde2 in regulating the cAMP/PKA pathway, we generated *pde1*Δ, *pde2*Δ, and *pde1*Δ *pde2*Δ mutants and analyzed their phenotypic traits (Fig. S2). We additionally constructed complemented strains and validated the restoration of the wild-type phenotype (Fig. S2). Previous studies have reported that *PDE2* deletion decreases thermotolerance in *S. cerevisiae* and *Candida albicans*, whereas *PDE1* deletion has no effect (25, 26). Consistent with these findings, we observed that *pde1*Δ was as thermotolerant as the wild type, whereas *pde2*Δ showed markedly decreased thermotolerance compared to the wild type (Fig. 2A and B). The *pde1*Δ *pde2*Δ mutant was even more defective in thermotolerance than *pde2*Δ (Fig. 2A). Moreover, we found that *pde2*Δ and *pde1*Δ *pde2*Δ mutants were unable to grow at 43°C (Fig. 2B). We confirmed that heat shock at 42°C for 2 h increased *PDE1* and *PDE2* expression relative to the basal condition of 30°C (Fig. S3). We also measured the ability of the mutants to recover from heat stress (at 50°C and 53°C for 10 min) and found that *pde1*Δ recovered to a similar extent as the wild type, whereas *pde2*Δ failed to recover properly. The *pde1*Δ *pde2*Δ mutant recovered more poorly than *pde2*Δ (Fig. S3). The heat shock-sensitive phenotype of *pde2*Δ was similar to that of *bcy1*Δ, which has a hyperactivated cAMP/PKA pathway (Fig. S3).

We next investigated whether PDEs regulate the expression levels of major heat shock proteins involved in thermotolerance. Under heat shock conditions, the expression of HSP genes was more strongly induced in *pde2*Δ and *pde1*Δ *pde2*Δ mutants than in the wild type or *bcy1*Δ and *pde1*Δ strains. The expression of *HSP60*, *HSP78*, and *HSP90* was induced by approximately threefold to fourfold in *pde2*Δ and *pde1*Δ *pde2*Δ mutant strains relative to wild type or *bcy1*Δ and *pde1*Δ strains (Fig. 2C), suggesting that the increased thermosensitivity of PDE mutants may be compensated by induction of HSP genes.

To confirm whether Bcy1 acts downstream of PDEs, we overexpressed *BCY1* in the *pde1*Δ *pde2*Δ background using the constitutively active *TEF1* promoter (Fig. S3). *BCY1* overexpression restored thermotolerance and several other stress-resistant phenotypes in *pde1*Δ *pde2*Δ (Fig. 2D), suggesting that Pde1 and Pde2 play minor and major roles, respectively, in negatively regulating the Ras/cAMP/PKA signaling pathway, and their deletion can decrease the thermotolerance of *C. auris*.

### Hyperactivated cAMP/PKA pathway decreases the survival rate of *C. auris* under nutrient-starved conditions

A hallmark of fungal cells with a hyperactivated cAMP/PKA pathway is a reduction in intracellular glycogen levels (29, 30). Glycogen is crucial as a carbon and energy source and serves as the primary reservoir of glucose in yeast cells (31). Pathogenic fungi typically reside in nutrient-poor environments, such as the host’s skin surface and organs, where the conversion of glucose into glycogen and its storage are essential for their long-term survival (32).

**Fig 3:**
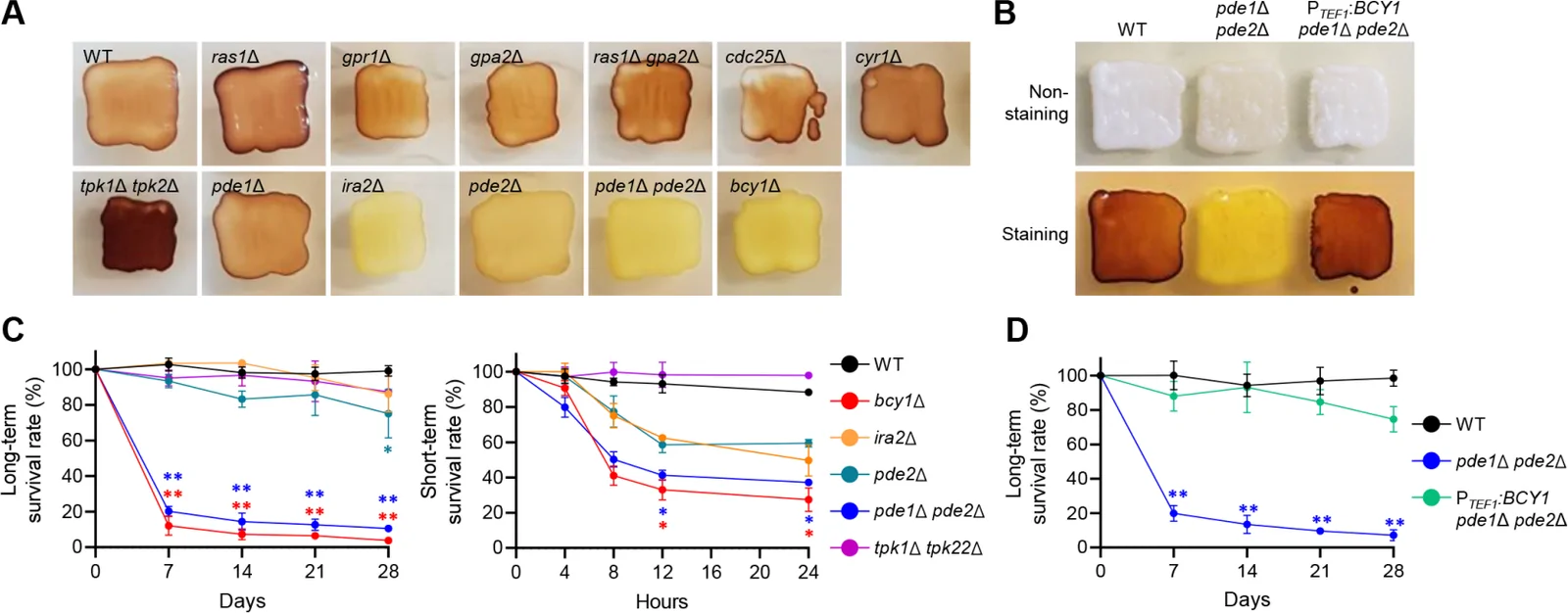
The role of Ras/cAMP/PKA signaling pathway in nutritional susceptibility of *C. auris*. (A and B) Glycogen accumulation in wild type and Ras/cAMP/PKA mutants. (C and D) The survival rate of *C. auris* wild type and mutants in nutrient starvation conditions. Statistical analysis was performed using one-way ANOVA with Bonferroni’s multiple-comparison test (*P < 0.05 and **P < 0.01).

To evaluate glycogen accumulation, we streaked wild type and mutant strains onto solid yeast extract-peptone-dextrose (YPD) media and incubated them at 30°C for 2 days, followed by staining the colonies with an iodine/iodide solution (Fig. 3A). The Ras/cAMP/PKA inactivated mutants generally exhibited darker brown staining than the wild type, with *tpk1*Δ *tpk2*Δ exhibiting the darkest brown staining. Conversely, Ras/cAMP/PKA hyperactivated mutants, such as *bcy1*Δ, *ira2*Δ, *pde2*Δ, and *pde1*Δ *pde2*Δ, did not show brown staining (Fig. 3A). Additionally, *BCY1* overexpression restored wild-type levels of intracellular glycogen in *pde1*Δ *pde2*Δ (Fig. 3B). These results suggest that the hyperactivation of the Ras/cAMP/PKA pathway in *C. auris* reduced glycogen accumulation.

We then assessed how altered glycogen accumulation levels affect the survival rate of *C. auris* under nutrient-starved conditions. To measure the long-term survival rate, we spotted wild-type and mutant strains onto solid nutrient-deprived media (yeast nitrogen base [YNB] without ammonium sulfate and amino acid) and incubated plates at 25°C for 28 days, assessing viability every 7 days. The survival rates of *bcy1*Δ and *pde1*Δ *pde2*Δ mutants declined sharply in the first 7 days, followed by a gradual decline over the next 21 days, with only 3.7% of *bcy1*Δ and 10.5% of *pde1*Δ *pde2*Δ mutants surviving at the end of the 28-day incubation period (Fig. 3C). The *pde2*Δ mutant showed a significant decrease, with only 75% of the strains surviving, while the *ira2*Δ mutant showed no statistically significant decrease with 87% of the strains surviving (Fig. 3C). For short-term survival rate measurement, wild type and each mutant were grown to the stationary phase in liquid nutrient-deprived medium at 30°C for 2 days and subsequently resuspended in phosphate-buffered saline (PBS). The results indicated that *bcy1*Δ and *pde1*Δ *pde2*Δ mutants were more vulnerable to nutrient deprivation than both wild-type and other mutant strains (Fig. 3C). Following inoculation in PBS, *bcy1*Δ, and *pde1*Δ *pde2*Δ mutants exhibited a rapid decline in survival rates, with only 27.4% and 37.2% of the strains surviving after 24 h, respectively (Fig. 3C). The survival rates of *pde2*Δ and *ira2*Δ mutants were 59.2% and 49.7%, respectively, after 24-h incubation in PBS, but the decrease was not statistically significant (Fig. 3C). *BCY1* overexpression completely restored the wild-type survival rate in *pde1*Δ *pde2*Δ under nutrient-starved conditions (Fig. 3D), consistent with the data shown in Fig. 3B. These findings suggest that the Ras/cAMP/PKA signaling pathway plays a crucial role in regulating intracellular glycogen accumulation and sensitivity to nutrient starvation in *C. auris*.

### Hyperactivation of the Ras/cAMP/PKA signaling pathway leads to increased biofilm formation ability of *C. auris*

**Fig 4:**
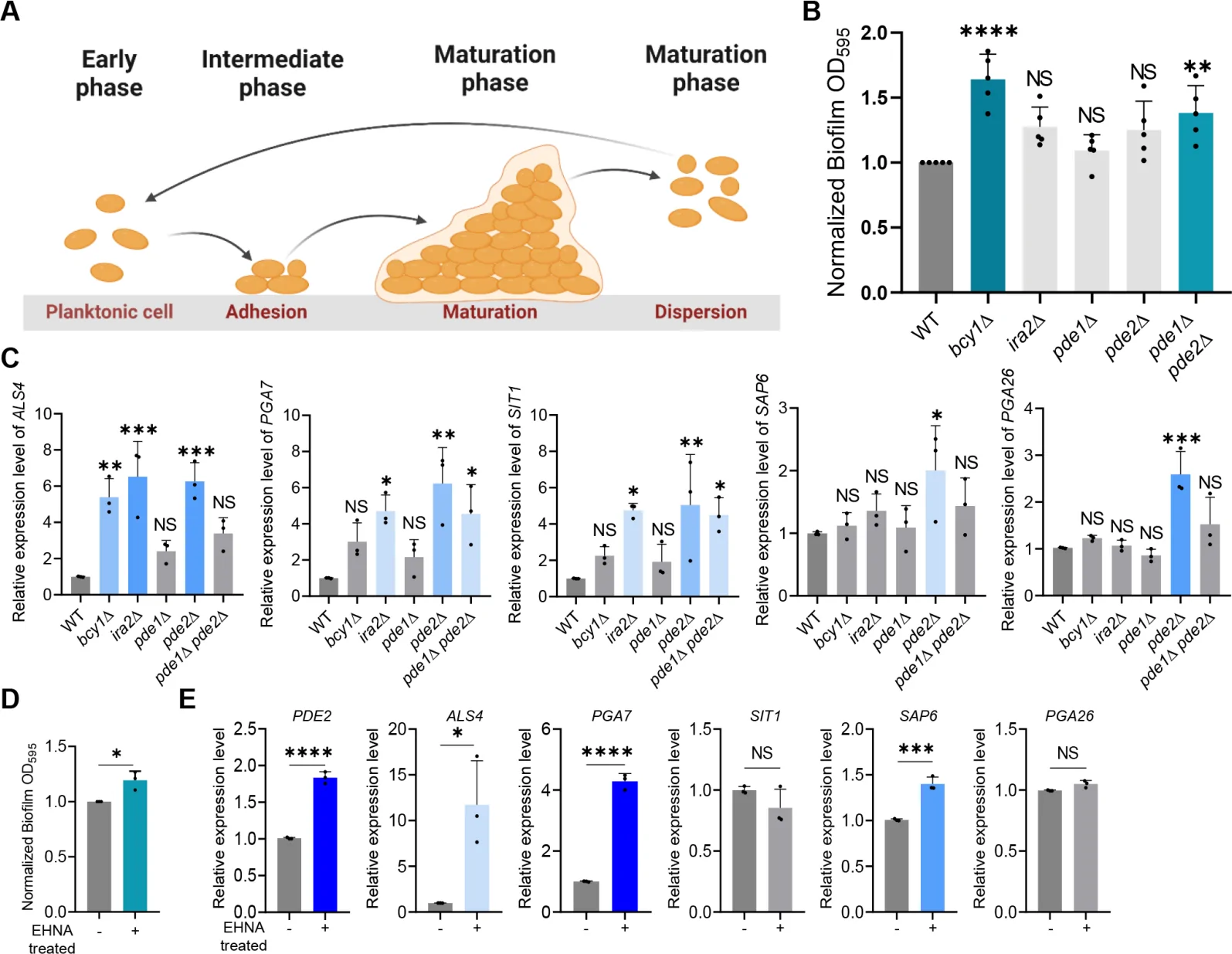
The role of Ras/cAMP/PKA signaling pathway in biofilm formation of *C. auris*. (A) Schematic representation of biofilm development phases in *Candida* species. (B) Biofilm formation assay of *C. auris* wild type and mutants. (C) qRT-PCR analysis of cell adhesion-related genes in wild type and mutants. (B and C) Statistical analysis was performed using one-way ANOVA with Bonferroni’s multiple-comparison test (*P < 0.05, **P < 0.01, ***P < 0.001, and ****P < 0.0001). (D) Biofilm formation assay of *C. auris* wild type with EHNA [erythro-9-(2-hydroxy-3-nonly)adenine] treatment. (E) qRT-PCR analysis of cell adhesion-related genes in wild type with EHNA treatment. (D and E) Statistical analysis was performed using Student’s *t* test (*P < 0.05, **P < 0.01, ***P < 0.001, and ****P < 0.0001).

Biofilm formation is a crucial component of virulence for *Candida* and other pathogenic fungi, as it confers resistance to antifungal medications and environmental stresses (33 – 35). The process of biofilm formation involves the adhesion of planktonic cells to solid surfaces, followed by biofilm maturation and dispersal of cells to form new biofilms (Fig. 4A). Previous research has shown that the *bcy1*Δ mutant has an enhanced capacity for biofilm formation relative to the wild type, whereas the *tpk1*Δ *tpk2*Δ mutant has a decreased biofilm capacity (21). We found that *pde1*Δ *pde2*Δ exhibited increased biofilm formation ability compared to the wild type (Fig. 4B), suggesting that the Ras/cAMP/PKA pathway promotes the biofilm formation in *C. auris*.

We also investigated the expression of genes involved in cell adhesion, which is the initial stage of biofilm formation, namely *ALS4*, *PGA7*, *SIT1*, *SAP6*, and *PGA26* (36 – 39). Our results showed that *ALS4* was upregulated in *bcy1*Δ, *ira2*Δ, and *pde2*Δ, while *PGA7* and *SIT1* were significantly increased in *ira2*Δ, *pde2*Δ, and *pde1*Δ *pde2*Δ (Fig. 4C). We observed an antagonistic effect of Pde1 and Pde2, as the expression levels of *ALS4*, *PGA7*, and *SIT1* were consistently increased in *pde2*Δ but only slightly increased in *pde1*Δ with no statistical significance, leading to a relatively lower expression in *pde1*Δ *pde2*Δ compared to *pde2*Δ (Fig. 4C). However, the expression levels of *SAP6* and *PGA26*, which act as negative regulators, were enhanced in *pde2*Δ, which could explain why *pde2*Δ formed less biofilm than *pde1*Δ *pde2*Δ and other mutants. Our findings suggest that cell adhesion, promoted by the Ras/cAMP/PKA pathway, is critical for the biofilm formation in *C. auris*.

To further explore the effect of *PDE2* inhibition on biofilm formation, we treated the wild-type strain of *C. auris* with the *PDE2* inhibitor EHNA [erythro-9-(2-hydroxy-3-nonyl)adenine]. We found that treatment with 50 µM EHNA resulted in a 20% increase in biofilm formation compared to the nontreated wild-type control group (Fig. 4D). The expression of *PDE2* increased by 60% upon treatment with EHNA, indicating a compensating response to the inhibitor (Fig. 4E). The expression levels of genes involved in cell adhesion were also altered, with the positive regulator *ALS4* increasing approximately 10-fold and *PGA7* increasing approximately fourfold compared to the nontreated wild-type group (Fig. 4E). However, *SIT1* did not show a significant difference, and the negative regulator *SAP6* increased by approximately 1.4-fold, while *PGA26* did not show a statistical difference (Fig. 4E). Our results suggest that hyperactivation of the Ras/cAMP/PKA pathway in *C. auris* can increase the expression levels of multiple genes associated with biofilm formation, leading to enhancing biofilm formation in *C. auris*.

### Hyperactivation of the Ras/cAMP/PKA signaling pathway attenuates the virulence of *C. auris*

**Fig 5:**
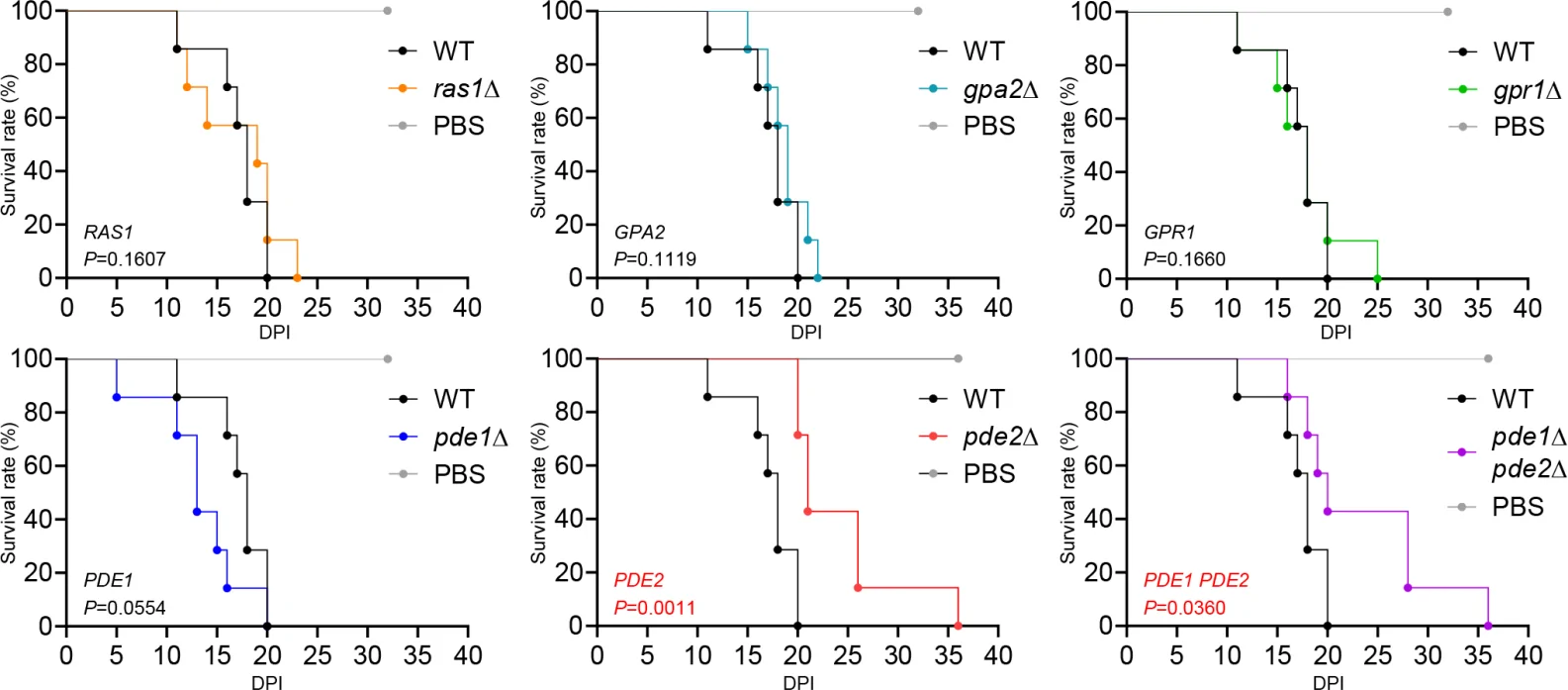
The role of Ras/cAMP/PKA signaling pathway in pathogenicity of *C. auris*. Female BALB/c mice (7-week-old) were injected intravenously with 1 × 10^7^ cells of strains of *C. auris* wild type and mutants. Survival was monitored daily, and statistical analysis was performed with Log-rank (Mantel-Cox) test by using Prism 8.0.

We established that disrupting the PKA regulatory subunit Bcy1 attenuates *C. auris* virulence, while disrupting the adenylate cyclase Cyr1 and PKA catalytic subunits Tpk1/2 does not (21). This suggests that the hyperactivated Ras/cAMP/PKA pathway can reduce *C. auris* virulence. We hypothesized that disrupting Pde1 and Pde2 would lead to decreased virulence, while disrupting Ras1, Gpa2, and Gpr1 would not affect *C. auris* virulence. The virulence of *ras1*Δ, *gpa2*Δ, *gpr1*Δ, and *pde1*Δ was similar to that of the wild-type strain, with mice inoculated with these strains dying mostly within 20–25 days of infection (Fig. 5). However, *pde2*Δ and *pde1*Δ *pde2*Δ exhibited reduced virulence compared to the wild type, with mice inoculated with these mutants surviving up to 16 days longer than those inoculated with the wild type (Fig. 5). These results support our hypothesis that hyperactivated Ras/cAMP/PKA pathway attenuates *C. auris* virulence.

## DISCUSSION

**Fig 6:**
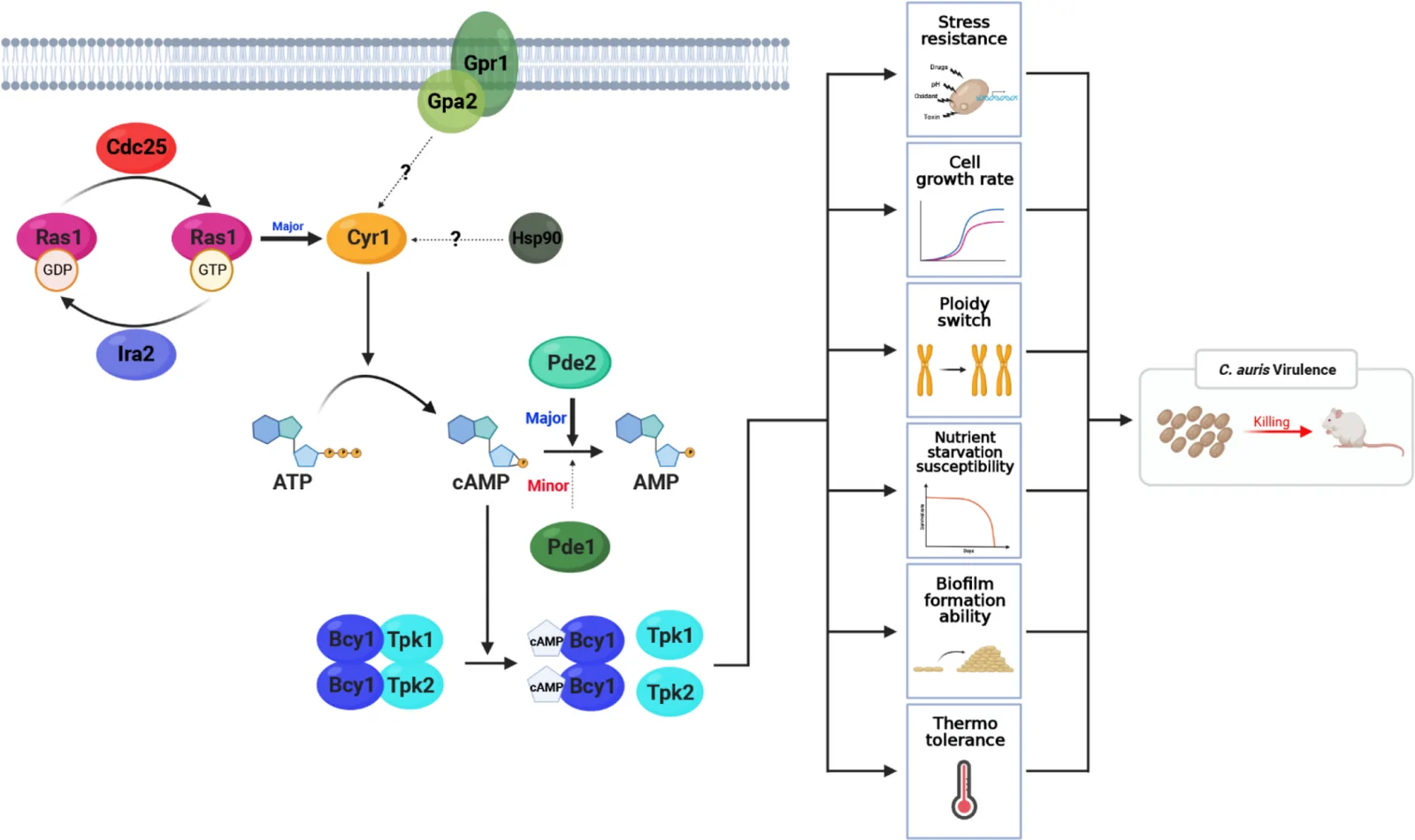
Proposed pathobiological functions and regulatory mechanisms of the Ras/cAMP/PKA signaling pathway in *C. auris*. The principal regulation of Cyr1 is conducted by Ras1, while cAMP degradation is handled by Pde1 and Pde2, serving minor and major roles, respectively. The Ras/cAMP/PKA pathway influences several phenotypes, including stress resistance, cell growth rate, ploidy change, nutrient starvation susceptibility, biofilm formation, and thermotolerance. These combined effects contribute to the virulence of *C. auris*. Notably, the inhibition of Bcy1 and Pde2 significantly heightens susceptibility to nutrient starvation and high temperature, underscoring their crucial role in *C. auris* pathogenicity.

In this study, we investigated the upstream signaling and negative feedback regulatory networks of the cAMP/PKA pathway required for the pathobiological process of *C. auris*. Our results demonstrate that the RAS signaling branch, consisting of Ras1, Cdc25, and Ira2, is a primary upstream regulator of the adenylyl cyclase Cyr1 in *C. auris*. In contrast, GPCR and other undefined signaling branches play minor roles. Regarding homeostatic regulation of the Ras/cAMP/PKA pathway, Pde2 and Pde1 are the primary and secondary regulators, respectively, that hydrolyze cAMP (Fig. 6). Furthermore, our findings indicate that hyperactivation of the Ras/cAMP/PKA signaling pathway can decrease the virulence of *C. auris*, while inactivation of the pathway only affects drug resistance and has no direct effect on virulence.

Fungal adenylyl cyclases contain multiple protein-interaction domains, including the Ras-associating and Gα-binding domains, and are primarily regulated by the GPCR and RAS signaling branches. Adenylyl cyclases in *C. albicans*, *S. cerevisiae*, and *Aspergillus fumigatus* contain both domains and are activated by GPCRs and Ras (Fig. S4). However, in *Cryptococcus neoformans*, the adenylyl cyclase Cac1 lacks the Ras-associating domain and is solely activated by the GPCR signaling branch (Fig. S4). In this study, we found that Cyr1 in *C. auris* is mainly activated by Ras1. Supporting it, we observed that *C. auris* Cyr1 lacks a Gα-binding domain, suggesting that it may not sense signals through GPCRs and Gα subunits (Fig. S4). As a result, *ras1*Δ and *cyr1*Δ displayed similar phenotypes, while *gpr1*Δ and *gpa2*Δ were phenotypically distinct from *cyr1*Δ. The divergent upstream signaling branches of the cAMP/PKA pathways among fungi may reflect their need to respond and adapt to corresponding biological niches. In *C. albicans*, known external cues for Ras1 activation include serum, amino acids, and *N*-acetyl glucosamine (40, 41). Although our data indicate that *C. auris* Ras1 may sense various stress signals, the exact external cues remain to be elucidated in future studies.

Our study suggests the presence of an alternative pathway that activates Cyr1 in *C. auris*, which is distinct from Ras and GPCR signaling. This is evident from our observation that *cyr1*Δ mutants exhibit more severe phenotypes than *ras1*Δ *gpa2*Δ double mutants. One possible candidate for this alternative pathway is an Hsp90-dependent signaling pathway. In *C. albicans*, the leucine-rich domain of Cyr1 interacts with Hsp90 to regulate temperature-dependent hyphal growth (41). Considering that *C. auris* Cyr1 also possesses this domain, it is reasonable to hypothesize that Hsp90 may serve as an additional upstream regulator of Cyr1 in *C. auris*. Supporting this idea, we found that *cyr1*Δ mutants were more sensitive to high temperature and heat shock than *ras1*Δ *gpa2*Δ. Another potential candidate includes other Ras-like small GTPases in *C. auris*. Our analysis of the *C. auris* genome revealed the presence of more than 20 small GTPases. Their role in the cAMP/PKA pathway should be further investigated in future studies.

The cAMP/PKA pathway in fungi requires negative feedback regulation to maintain biological balance. PDEs, which can hydrolyze cAMP to AMP, are key negative feedback regulators of the cAMP pathway in many fungal species. Ascomyctoa fungi, including *C. albicans*, *S. cerevisiae*, and *Aspergillus flavus*, primarily employ high-affinity PDEs (15, 26, 28). However, some Basidiomycota fungi, such as *C. neoformans*, use low-affinity PDEs as their primary regulator (27). In this study, we show that *C. auris*, an Ascomycota fungus, primarily employs high-affinity PDE (Pde2) as the primary negative regulator of the cAMP/PKA pathway, while low-affinity PDE (Pde1) plays a secondary role. Notably, *Ustilago maydis*, a Basidiomycota fungus, uses both high-affinity (Umpde1) and low-affinity (Umpde2) PDEs to regulate various biological processes, with Umpde1 being primarily required for virulence (42). Interestingly, heterologous expression of Umpde1 in *pde1*Δ mutants of *C. neoformans* and *pde1*Δ *pde2*Δ mutants of *S. cerevisiae* can restore their wild-type phenotypes (42), suggesting redundant roles for high-affinity and low-affinity PDEs across diverse fungal lineages. However, our study also revealed antagonistic actions between Pde1 and Pde2 in regulating the expression of some biofilm-related genes, such as *ALS4*, *SAP6*, and *PGA26*, in *C. auris*. Therefore, further investigation is needed to elucidate the redundant and distinct roles of these two PDEs in *C. auris*.

Our study revealed that hyperactivation of the Ras/cAMP/PKA pathway has both positive and negative effects on the pathogenicity of *C. auris*. Deletion of Bcy1, Ira2, Pde1, and Pde2 led to decreased glycogen accumulation and reduced survival under nutrient-starved conditions, which may reduce the virulence of *C. auris*. Previous studies have demonstrated that deletion of PDEs in *C. albicans* leads to impaired glycogen accumulation, reduced survival rates under nutrient-starved conditions, and attenuated virulence (29). A similar observation was also made in *A. flavus* (28). The mammalian host environment is characterized by nutrient scarcity, affecting the competitive fitness of a pathogen with an impaired accumulation of glycogen as a carbon source. In addition, the thermotolerance of *bcy1*Δ, *pde2*Δ, and *pde1*Δ *pde2*Δ mutants was significantly reduced, which may also decrease the virulence. In contrast, hyperactivation of the Ras/cAMP/PKA pathway by deletion of Bcy1, Ira2, Pde1, or Pde2 increased biofilm formation, which could enhance the virulence of *C. auris,* because cells in biofilm could be more tolerant to the host immune system (43). Therefore, these compounding effects by hyperactivation of the Ras/cAMP/PKA pathway may lead to the less pronounced virulence attenuation in *pde2*Δ and *pde1*Δ *pde2*Δ mutants.

In conclusion, we have comprehensively analyzed the Ras/cAMP/PKA pathway and established its role in various aspects of *C. auris* pathobiology. We have demonstrated that hyperactivation of this pathway is linked to key virulence factors, such as thermotolerance, biofilm formation, and nutrient susceptibility. Although the specific functions of the Ras/cAMP/PKA pathway may vary depending on the clade, its importance in the various pathobiological functions of *C. auris* is widely recognized. Therefore, both aspects of this pathway must be taken into account when developing treatments for patients infected with *C. auris*. One promising approach is to combine an Ras/cAMP/PKA pathway inhibitor with existing antifungal drugs. Although inactivation of this pathway may increase resistance to AMB, it can significantly reduce resistance to other drugs such as azoles or echinocandins. Therefore, combination therapy with a PKA inhibitor is expected to be highly effective in treating *C. auris* infections. A second strategy involves using a Pde2 inhibitor to hyperactivate the Ras/cAMP/PKA pathway. Although it may lead to increased biofilm formation, it could significantly reduce thermotolerance and glycogen accumulation, resulting in decreased virulence in the host. By considering both of these methods, we can more effectively treat patients infected with *C. auris* in the future.

## MATERIALS AND METHODS

### *Candida auris* strains and growth media

*Candida auris* strains used in this study are listed in Table S1 in the supplemental material. The parental wild-type strain, B8441 (AR0387), was obtained from the CDC. These isolates and the constructed mutant strains were stored as frozen stocks in 20% glycerol at −80°C until further use. Yeast strains were routinely cultured on YPD agar plates (2% agar in YPD broth: 1% yeast extract, 2% peptone, and 2% D-glucose) at 30°C for 24–48 h. For liquid cultures, cells were grown in YPD broth at 30°C with shaking at 200 rpm. For experimental assays, cells were inoculated into fresh YPD broth and grown to mid-log phase (optical density at 600 nm [OD_600_] = 0.6–0.8) before being subjected to various treatments.

### Gene deletion and complementation

To generate gene deletion mutants, we used the nourseothricin resistance marker (*CaNAT*), hygromycin B resistance marker (*CaHYG*), or G481 resistance marker (*CaNEO*) flanked by 0.5–1.0 kb 5′ and 3′ regions of each target gene, including *RAS1*, *GPR1*, *GPA2*, *CDC25*, *IRA2*, *PDE1*, and *PDE2*. Each gene disruption cassette containing a selection marker was constructed using double-joint PCR. To amplify the flanking regions of a target gene, we used L1-L2, and R1-R2 primer pairs in the first round of PCR. The *CaNAT* selection marker was amplified by PCR using the plasmid pV1025 containing the *CaNAT* gene as a template and the primer pairs listed in Table S1 in the supplemental material. The first round of PCR products of the flanking regions and *CaNAT* marker were purified together and used as templates for the second round of double-joint PCR. In the second round of PCR, 5′- and 3′-gene disruption cassettes containing split *CaNAT* selection markers were amplified by L1-split primer 2 and R2-split primer 1, respectively (Table S1 in the supplemental material).

For the transformation of *C. auris* with gene disruption cassettes, we used a lithium acetate/heat-shock protocol with modifications. Cells were cultured overnight at 30°C in 50 mL YPD broth with shaking. We centrifuged 1.2 mL of cultured cells, washed them with dH_2_O and lithium acetate buffer (100 mM lithium acetate, 10 mM Tris, 1 mM EDTA, pH 7.5), and resuspended them in 300 µL of lithium acetate buffer. The transformation was set up with 10 µL of denatured salmon sperm DNA (Sigma, cat no. D9156), 100 µL of the competent cells, 500 µL of 50% PEG4000 (Sigma, cat no. P4338), and 50 µL of the amplified gene deletion cassette. The transformation mixture was incubated at 30°C for 6 h with intermittent vortexing. Subsequently, the cells were subjected to a 20-min heat shock at 42°C followed by 1 min of cooling on ice. The cells were then pelleted, resuspended in 1 mL of YPD medium, and incubated at 30°C for 1 h with shaking. After the incubation, the cells were washed twice with fresh liquid YPD medium and then spread onto selective YPD agar plates supplemented with 600 µg/mL nourseothricin, 1.8 mg/mL hygromycin B, or 2.4 mg/mL G418. The plates were then incubated at 37°C for 2–3 days. We confirmed the desired genotype of each positive nourseothricin-, hygromycin B-, or G418-resistant transformant by diagnostic PCR and Southern blot (Fig. S2).

To confirm the phenotypes of the *ras1*Δ, *gpr1*Δ, *gpa2*Δ, *cdc25*Δ, *ira2*Δ, *pde1*Δ, and *pde2*Δ mutants, we constructed corresponding complemented strains, in which each wild-type allele was either ectopically integrated (*ras1*Δ + *RAS1, gpa2*Δ + *GPA2*, and *cdc25*Δ + *CDC25*) or re-integrated into its native locus (*gpr1*Δ::*GPR1, pde1*Δ::*PDE1, pde2*Δ::*PDE2*, and *ira2*Δ::*IRA2*). To generate each full-length gene fragment, Phusion-PCR was performed using genomic DNA from the wild-type B8441 strain as a template and each primer pair listed in Table S1 in the supplemental material. The resulting fragments were directly cloned into the TOPO vector (Invitrogen) to generate the plasmids pTOP-RAS1, pTOP-GPR1, pTOP-GPA2, pTOP-CDC25, pTOP-IRA2, pTOP-PDE1, and pTOP-PDE2. After confirming the target sequence, the *CaHYG* inserts were sub-cloned into each pTOP vector to produce the pTOP-RAS1-HYG, pTOP-GPR1-HYG, pTOP-GPA2-HYG, pTOP-CDC25-HYG, pTOP-IRA2-HYG, pTOP-PDE1-HYG, and pTOP-PDE2-HYG. For the targeted re-integration into its native locus, pTOP-RAS1-HYG, pTOP-GPR1-HYG, pTOP-GPA2-HYG, pTOP-CDC25-HYG, pTOP-IRA2-HYG, pTOP-PDE1-HYG, and pTOP-PDE2-HYG were linearized by NsiI, BsmI, AfeI, AvrII, PmlI, AfeI, and AgeI, respectively, and introduced into each mutant by the lithium acetate heat-shock method. The correct genotype of the complemented strain was confirmed by diagnostic PCR (Fig. S2).

### Total RNA preparation and quantitative RT-PCR

Total RNA was extracted from *C. auris* wild-type and Ras/cAMP/PKA mutant strains cultured overnight at 30°C in YPD broth. Briefly, cells were collected by centrifugation after reaching an OD_600_ of 0.6–0.8, frozen in liquid nitrogen, and lyophilized. For stress conditions, 10 mL of the culture was sampled for the basal state, and the remaining 30 mL was further incubated with stress agents. Total RNA was isolated by the Trizol extraction method with Easy-blue (Intron). Complementary DNA was synthesized from purified total RNA using reverse transcriptase (Thermo Scientific). Quantitative PCR was performed using specific primer pairs for each gene and the CFX96TM Real-Time system (Bio-Rad). *ACT1* expression was used for normalization. Statistical analysis was performed using one-way ANOVA, followed by Bonferroni’s multiple-comparison test. All experiments were conducted in triplicate and repeated thrice biologically.

### Growth and stress sensitivity spot assay

To assess the growth and stress sensitivity of wild type and Ras/cAMP/PKA mutant strains of *C. auris*, cells were grown overnight at 30°C and serially diluted 10-fold, four times (final dilution 1:10^4^). The diluted cells were spotted onto YPD plates, which were then incubated at various temperatures (30°C, 37°C, 42°C, and 43°C). Growth was qualitatively assessed by photographing the plates after 1 day. To impose various stresses on the cells, different chemical agents were added to the media, including sorbitol (osmotic stress agent), NaCl or KCl (cation and salt stress), hydrogen peroxide, *tert*-butyl hydroperoxide, MD, or DIA (oxidative stress), SDS (membrane destabilizing stress), congo red (cell-wall destabilizing stress), and various antifungal agents (fludioxonil, flucytosine, FCZ, itraconazole, PCZ, caspofungin, or AMB). Cells were incubated at 30°C and photographed for 3 days after stress treatment.

### Construction of *BCY1* overexpression strains

To generate the *BCY1* constitutive overexpression strain, we replaced the native promoter of *BCY1* with the *TEF1* promoter using an amplified homologous recombination cassette (Fig. S3). In the first round of PCR, we amplified the native promoter and 5′ coding regions (from ATG) of *BCY1* using primer pairs B17979/B17980 and B17981/B17982, respectively. We also amplified the G418 resistance gene (NEO)-TEF1 promoter region with the primer pair B16376/B17918. In the second-round PCR, we used double joint-PCR to amplify the 5′ region of the P*_TEF1_:BCY1* cassette using the mixed templates of the native promoter region of *BCY1* and the 5′ region of the NEO-TEF1 promoter with the primer pair B17979/B16379 (Table S1). We similarly amplified the 3′ region of the P*_TEF1_:BCY1* cassette using the mixed templates of the 5′ coding region of *BCY1* and the 3′ region of the NEO-TEF1 promoter with the primer pair B16378/B17982. Next, we then introduced the combined split P*_TEF1_:BCY1* cassettes into the *pde1*Δ *pde2*Δ (YSBA70) strain by heat shock transformation. Stable transformants were selected on a YPD medium containing 2.4 mg/mL G418. Positive transformants were confirmed using diagnostic PCR with a primer pair B10742/B16380.

### Glycogen accumulation measurement assay

To measure the glycogen levels in each strain, iodine/iodide staining was performed as follows. All *C. auris* strains were streaked onto YPD agar plates and incubated at 30°C for 2 days. The colonies were then gently covered with 1 mL of Lugol solution (Sigma, cat no: 62650), and the plates were photographed 2 min later to visualize the iodine/iodide staining pattern.

### Survival rate measurement

To measure the long-term survival rate, *C. auris* strains were cultured overnight in 2 mL YPD broth at 30°C, washed twice with dH_2_O, spotted onto solid YNB media lacking amino acids and ammonium sulfate, and incubated at 25°C for 28 days. Every 7 days, cells were collected from the plate, resuspended in dH_2_O, and the cell count was determined. Approximately 300 cells were then spread onto a solid YPD medium, and the percentage survival rate was calculated at each time point using the formula: survival rate = (number of colonies at each time point/number of colonies on the control plate) × 100.

For short-term survival rate measurement, strains were grown to stationary phase in liquid YNB medium lacking amino acids and ammonium sulfate at 30°C for 2 days, washed, resuspended in 1× PBS at a concentration of 10^4^ cells/mL, and incubated at 25°C for 24 h. Aliquots were taken at 0, 4, 8, 12, 16, and 24 h of incubation, and approximately 300 cells were spread onto YPD plates. The plates were incubated at 37°C for 24 h, and the number of colonies was counted. The percentage survival rate of each strain at each time point was calculated as described above.

### Biofilm assay

To evaluate the biofilm formation of wild-type and Ras/cAMP/PKA mutant strains of *C. auris*, cells were cultured overnight in 2 mL YPD broth at 30°C. Cells were then suspended in RPMI-1640 media to an OD_600_ of 0.5, and 200 µL of the suspension was added to flat-bottomed 96-well plates. To reduce evaporation and prevent cross-contamination between wells, plates were sealed with Breathe-Easy sealing membranes. Plates were incubated at 37°C with shaking at 250 rpm for 90 min. The RPMI medium was then removed, and the wells were washed with 200 µL PBS before adding 200 µL of fresh RPMI-1640 medium. The plates were then re-sealed and incubated at 37°C with shaking at 250 rpm for 24 h. After incubation, the RPMI-1640 medium was removed, and OD_595_ was measured using a microplate reader. The average density of adhered cells was calculated from the optical density readings from three independent wells in the plate. Data were normalized by subtracting the average blank well value from the experimental and control well values, and then dividing the resulting value by the mean blank-subtracted OD_595_ for the control wells.

### Virulence study

To evaluate the virulence of *C. auris* wild type and Ras/cAMP/PKA mutant strains *in vivo*, we used a murine systemic infection model with reference to previous studies (21, 44). Fungal cells were incubated overnight at 30°C in YPD broth and washed three times with PBS. Cell concentrations were measured and adjusted to 10^8^ cells/mL in PBS. To confirm CFUs and viability of the inoculum, the diluted cells were plated onto YPD agar plates and incubated at 37°C for 24 h. Specific pathogen-free/viral antibody-free (SPF/VAF) inbred 6-week-old female mice of the BALB/c (AnNCrlOri) strain were used for this study (Orient Bio Inc., South Korea), and they were habituated for 1 week before the experiment. For infection, the mice were restrained, and their tails were placed in warm (40°C) water to expand the lateral veins. The 100 µL of cell suspension was injected intravenously. Daily monitoring of survival was performed and abnormal behavior (head tilt or body spinning) was judged as a symptom of infection (44), and the mice were sacrificed as a humane endpoint for the experiment. The survival curves were analyzed using the Log-rank (Mantel-Cox).

## Data Availability

We will provide any strain and materials used in this study upon request.

## SUPPLEMENTAL MATERIAL

Supplemental figures spectrum.02152-23-s0001.pdf Figures S1 to S4.

Table S1 spectrum.02152-23-s0002.pdf
*C. auris* strains and primers used in this study.

ASM does not own the copyrights to Supplemental Material that may be linked to, or accessed through, an article. The authors have granted ASM a non-exclusive, world-wide license to publish the Supplemental Material files. Please contact the corresponding author directly for reuse.
