# Supplementary material for: Deciphering the regulatory mechanisms of the cAMP/protein kinase A pathway and their roles in the pathogenicity of Candida auris
Source: Microbiol Spectr. 2023 Sep 6;11(5):e02152-23. doi: 10.1128/spectrum.02152-23 (PMC10581177; doi:10.1128/spectrum.02152-23)
Supplement: Supplemental figures — Figures S1 to S4. [file spectrum.02152-23-s0001.pdf]

Figure S1 (Kim et al.)

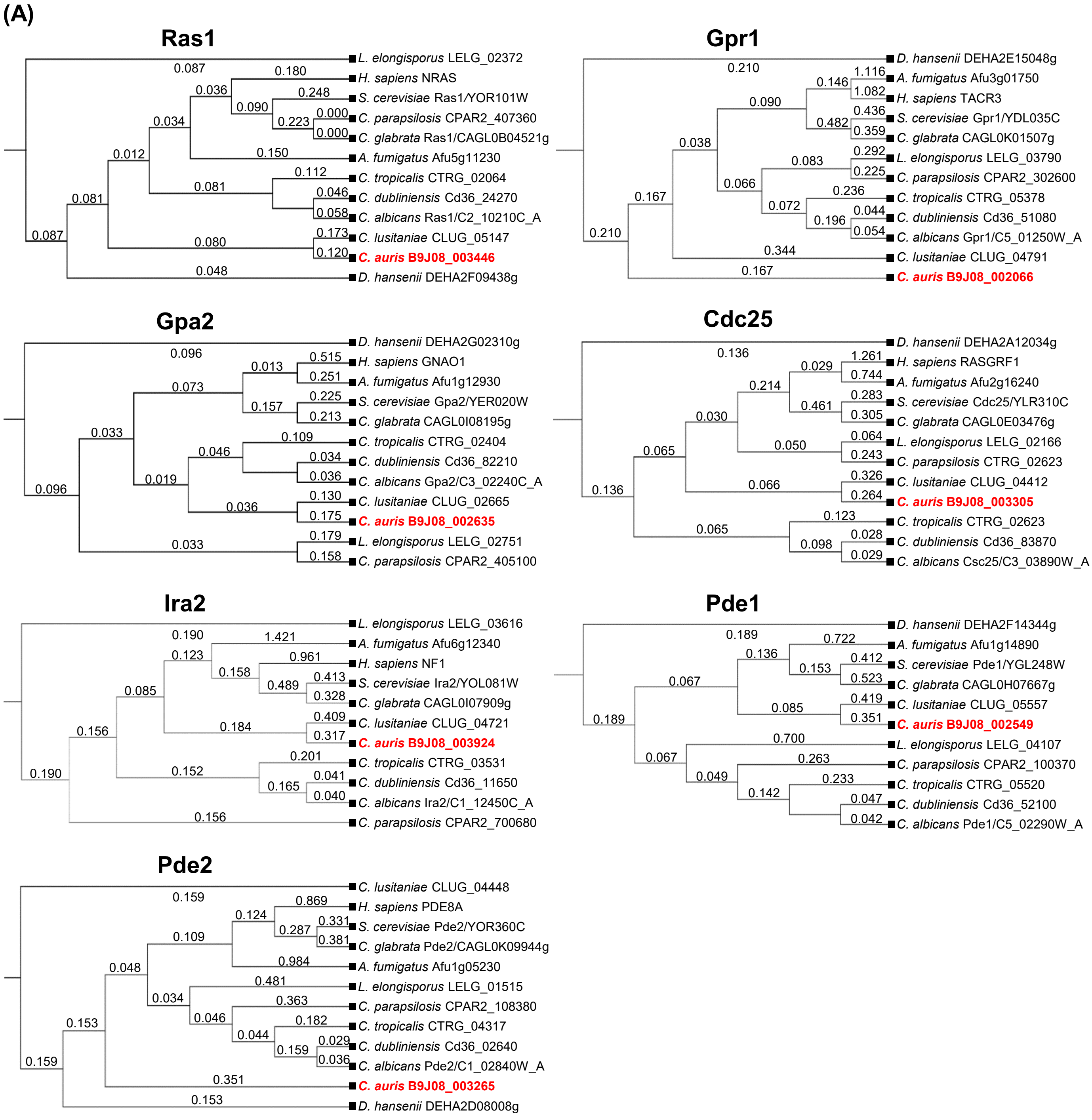

Figure S1 (Kim et al.)

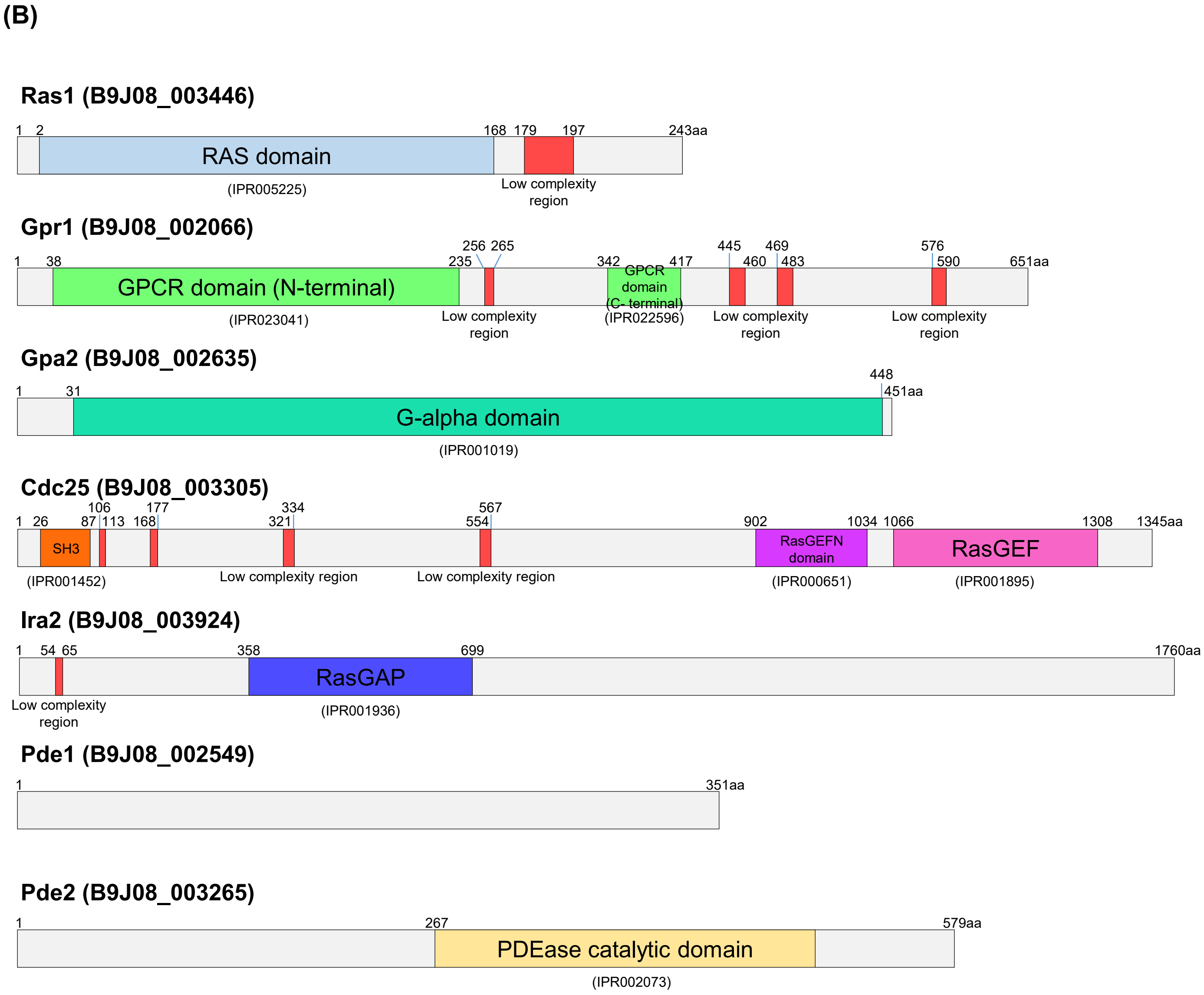

Figure S1. Evolutionary relationships and protein domains of Ras/cAMP/PKA signaling pathway genes. (A) A cladogram was constructed using the amino acid sequences of Ras1, Gpr1, Gpa2, Cdc25, Ira2, Pde1, and Pde2 from *Candida* species, related species, and humans. The analysis was performed using the CLC Sequence Viewer 8.0 software. (B) The protein domains of Ras/cAMP/PKA genes were analyzed using available domain information on InterPro (https://www.ebi.ac.uk/interpro/).

Figure S2 (Kim et al.)

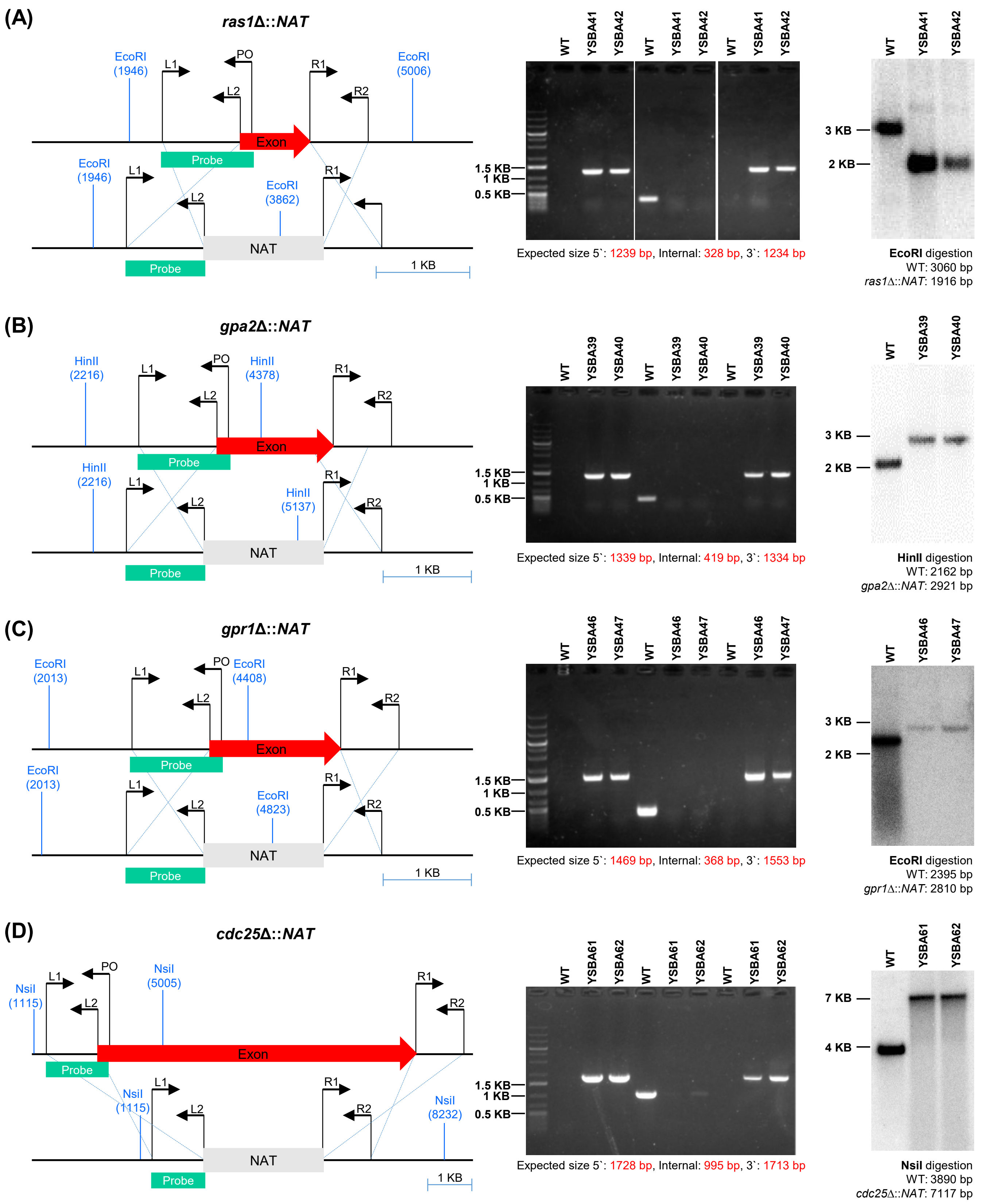

Figure S2 (Kim et al.)

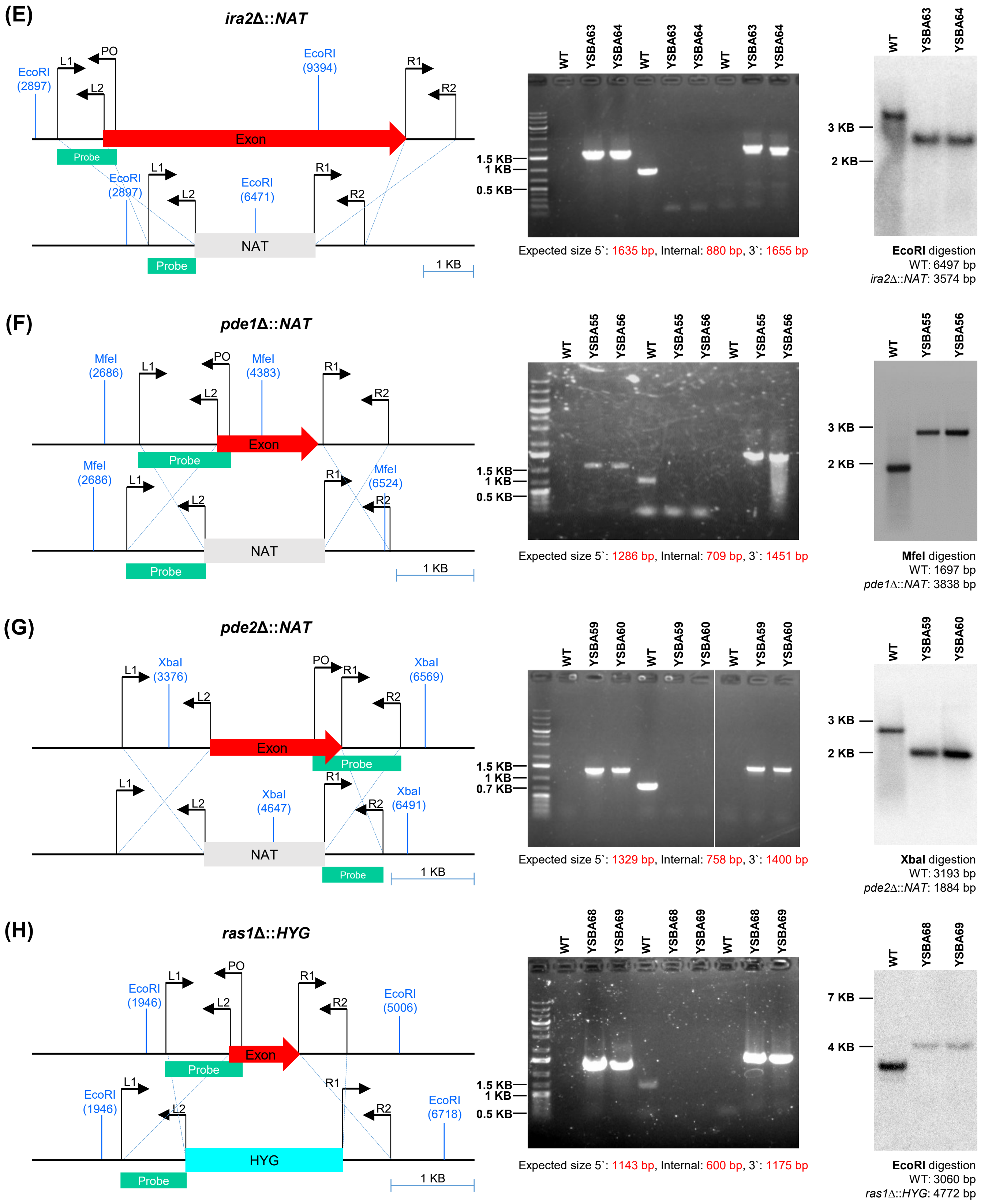

Figure S2 (Kim et al.)

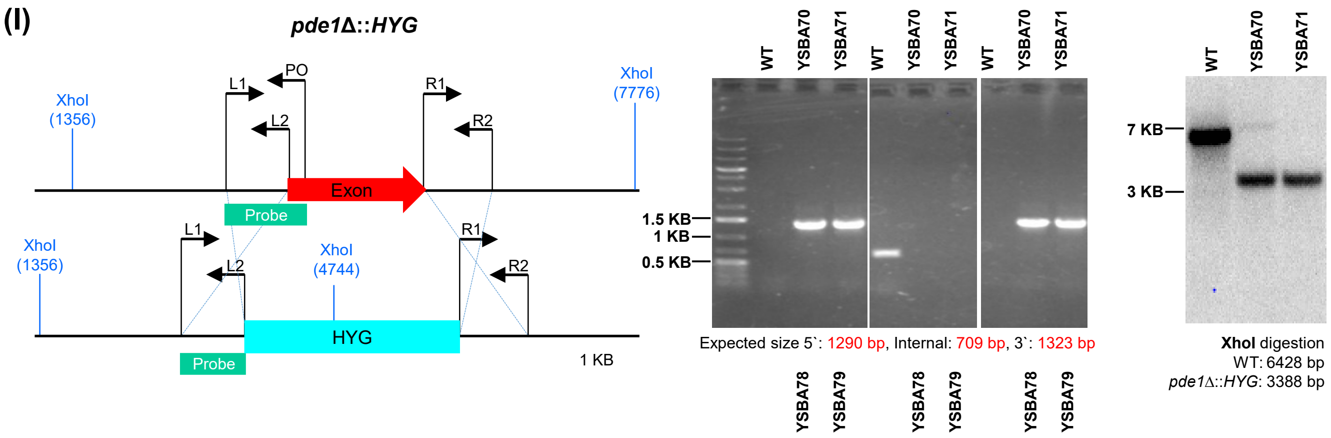

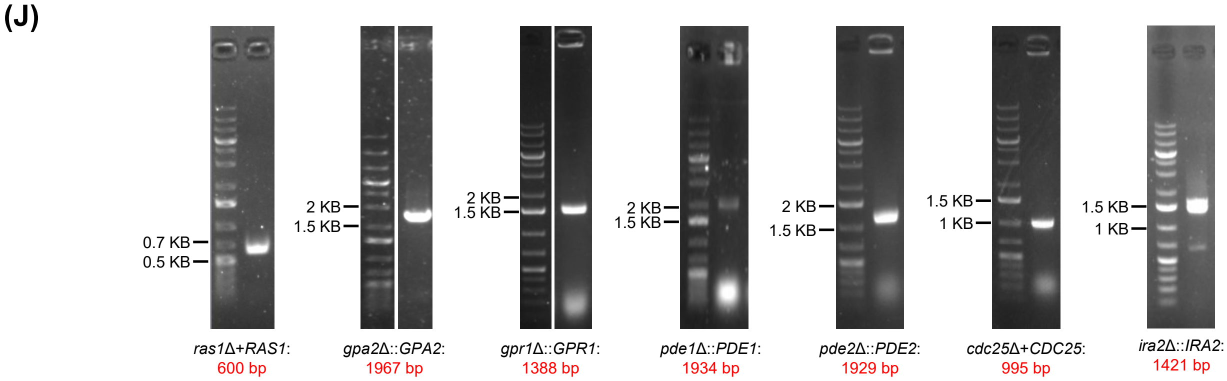

Figure S2 (Kim et al.)

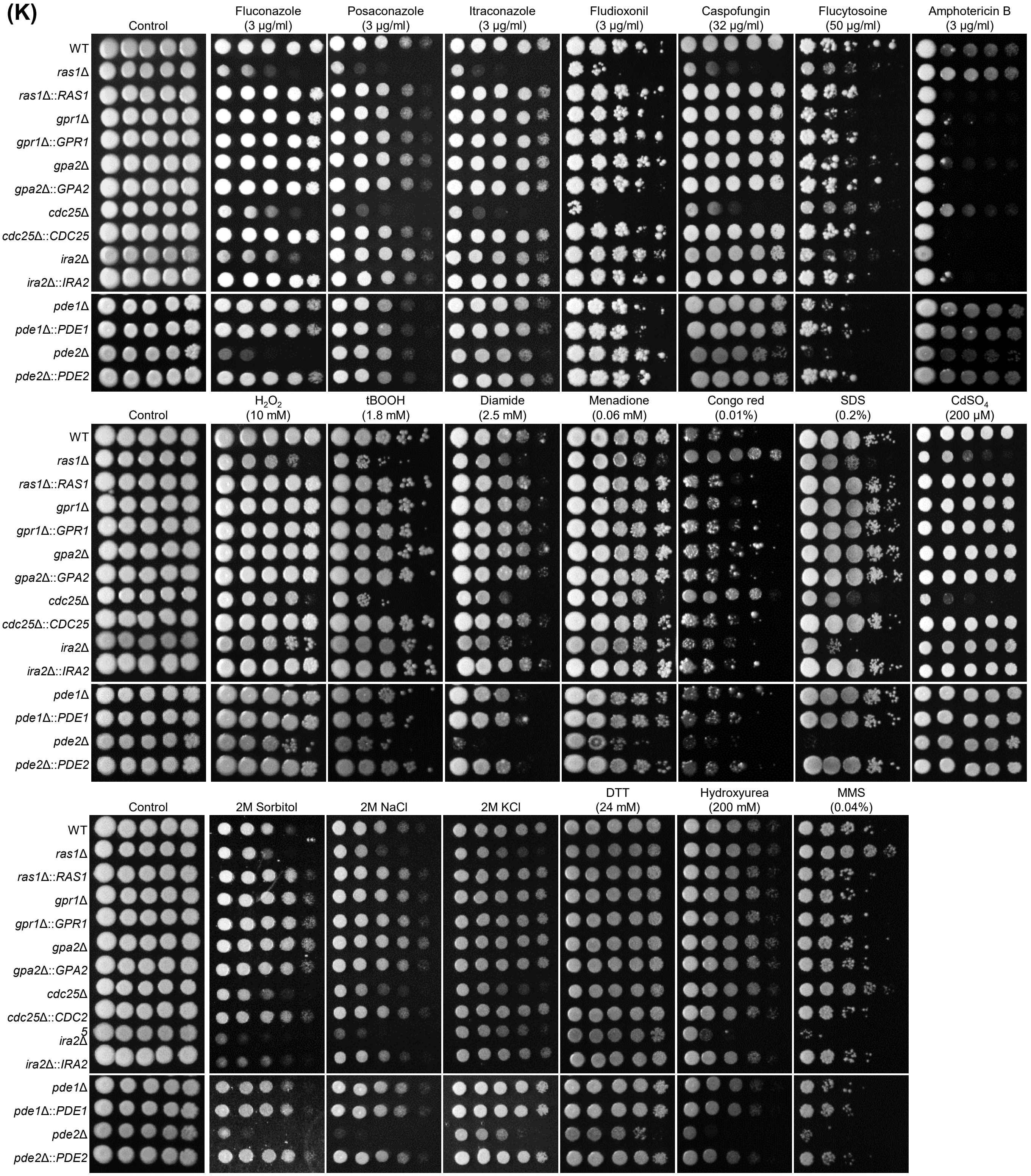

Figure S2. Construction, validation, and analysis of gene deletion mutants in *Candida auris*.
(A to I) Homologous recombination strategies between the wild-type gene and the deletion cassette are illustrated graphically (left panels). Transformants were confirmed by diagnostic PCR (center panels) and Southern blot analysis (right panels). (J) Validation of complemented strains. (K) Spot analysis of *C. auris* wild-type and mutants under stress-inducing conditions.

Figure S3 (Kim et al.)

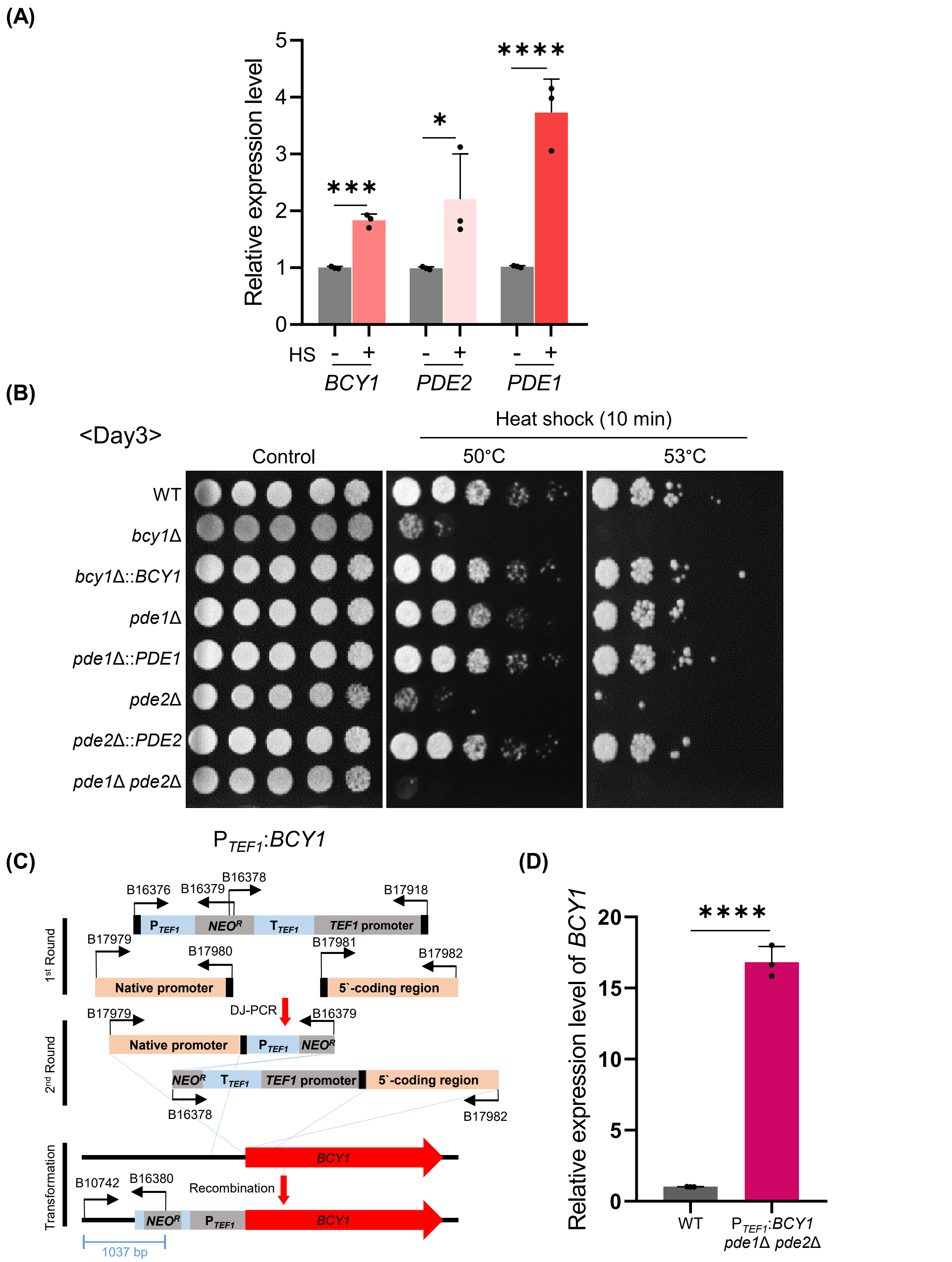

Figure S3. Validation of the roles of Pde1/2 and Bcy1 in *C. auris* thermotolerance. (A) qRT-PCR analysis of *BCY1*, *PDE1*, and *PDE2* in the wild-type strain. (B) Qualitative spot assays for measuring the thermotolerance of the wild-type and mutant strains. (C) Strategy for construction of the *BCY1* overexpression strain using the TEF1 promoter. (D) qRT-PCR analysis of *BCY1* in the wild-type and *BCY1*^{oe} *pde1Δ pde2Δ* strains. (A and D) Statistical analysis was performed using Student's t test (*, $P < 0.05$; **, $P < 0.01$; ***, $P < 0.001$; ****, $P < 0.0001$).

Figure S4 (Kim et al.)

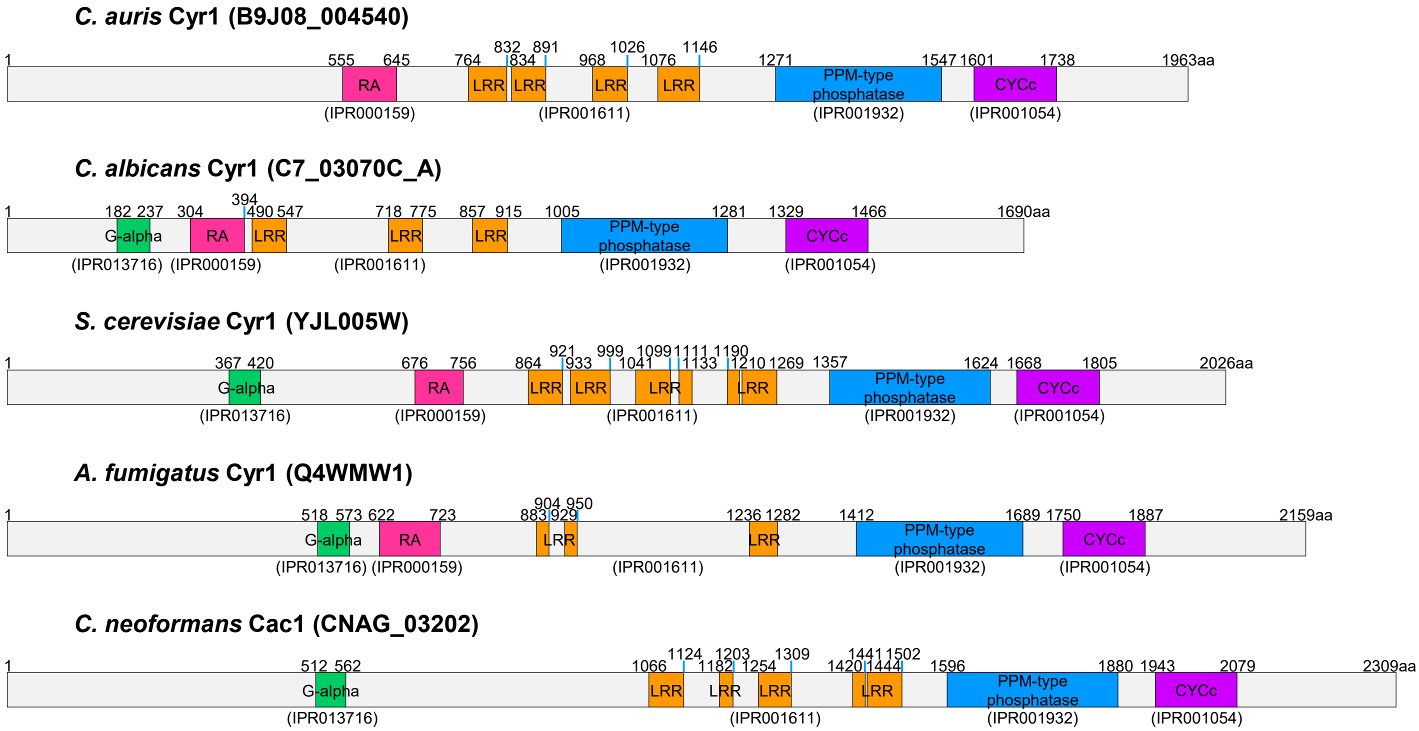

Figure S4. Protein domain analysis of adenylate cyclase in fungal species. The available domain information on InterPro (https://www.ebi.ac.uk/interpro/) was used to perform a protein domain analysis of adenylate cyclase in fungal species, such as *C. auris*, *C. albicans*, *S. cerevisiae*, *A. fumigatus*, and *C. neoformans*. (G-alpha: G-alpha binding domain, RA: Ras-associating domain, LRR: Leucine-rich repeat, CYCc: Adenylyl cyclase class-3/4/guanylyl cyclase domain).
