# Supplementary material for: Deciphering the regulatory mechanisms of the cAMP/protein kinase A pathway and their roles in the pathogenicity of Candida auris
Source: Microbiol Spectr. 2023 Sep 6;11(5):e02152-23. doi: 10.1128/spectrum.02152-23 (PMC10581177; doi:10.1128/spectrum.02152-23)
Supplement: Table S1 — C. auris strains and primers used in this study. [file spectrum.02152-23-s0002.pdf]

## Table S1. *C. auris* strains and primers used in this study

| **Strains used in this study** | | | |
|---|---|---|---|
| **Strain** | Genotype | **Parent** | **Reference** |
| **B8441** | Wild-type | | (1) |
| **YSBA4** | *bcy1*Δ::*NAT* | B8441 | (2) |
| **YSBA21** | *cyr1*Δ::*NAT* | B8441 | (2) |
| **YSBA24** | *tpk2*Δ::*NAT tpk1*Δ::*HYG* | YSBA17 | (2) |
| **YSBA39** | *gpa2*Δ::*NAT* | B8441 | This study |
| **YSBA40** | *gpa2*Δ::*NAT* | B8441 | This study |
| **YSBA41** | *ras1*Δ::*NAT* | B8441 | This study |
| **YSBA42** | *ras1*Δ::*NAT* | B8441 | This study |
| **YSBA46** | *gpr1*Δ::*NAT* | B8441 | This study |
| **YSBA47** | *gpr1*Δ::*NAT* | B8441 | This study |
| **YSBA50** | *ras1*Δ+*RAS1-HYG* | YSBA42 | This study |
| **YSBA53** | *gpa2*Δ+*GPA2-HYG* | YSBA39 | This study |
| **YSBA55** | *pde1*Δ::*NAT* | B8441 | This study |
| **YSBA56** | *pde1*Δ::*NAT* | B8441 | This study |
| **YSBA57** | *gpr1*Δ::*GPR1-HYG* | B8441 | This study |
| **YSBA59** | *pde2*Δ::*NAT* | B8441 | This study |
| **YSBA60** | *pde2*Δ::*NAT* | B8441 | This study |
| **YSBA61** | *cdc25*Δ::*NAT* | B8441 | This study |
| **YSBA62** | *cdc25*Δ::*NAT* | B8441 | This study |
| **YSBA63** | *ira2*Δ::*NAT* | B8441 | This study |
| **YSBA64** | *ira2*Δ::*NAT* | B8441 | This study |
| **YSBA66** | *pde1*Δ::*PDE1-HYG* | B8441 | This study |
| **YSBA68** | *gpa2*Δ::*NAT ras1*Δ::*HYG* | YSBA39 | This study |
| **YSBA69** | *gpa2*Δ::*NAT ras1*Δ::*HYG* | YSBA39 | This study |
| **YSBA70** | *pde2*Δ::*NAT pde1*Δ::*HYG* | YSBA59 | This study |
| **YSBA71** | *pde2*Δ::*NAT pde1*Δ::*HYG* | YSBA59 | This study |
| **YSBA93** | *cdc25*Δ+*CDC25-HYG* | YSBA61 | This study |
| **YSBA95** | *pde2*Δ::*PDE2-HYG* | YSBA70 | This study |
| **YSBA140** | *ira2*Δ::*IRA2-HYG* | YSBA63 | This study |
| **YSBA162** | $P_{TEF1}$:*BCY1*::*NEO pde2*Δ::*NAT pde1*Δ::*HYG* | YSBA70 | This study |

**Primers used in this study**

| Name | Primer description | Sequence (5’ to 3’) |
|---|---|---|
| **B13593** | *RAS1* 5’-flanking region primer L1 | GTGACGAGTGTTTGGCGAAG |
| **B13594** | *RAS1* 5’-flanking region primer L2 | TTCAGATCCACTAGTTCTAGAACTTATACTCTTTTAACAT |
| **B13595** | *RAS1* 3’-flanking region primer R1 | TTTCCAGAATTTCACTCTTAGGTGTTGTGTGATTGTGTAG |
| **B13596** | *RAS1* 3’-flanking region primer R2 | AAATGCGACAAACAAGAAAA |
| **B13597** | *RAS1* 5’-screening primer SO | TGATGGAGATTCGTGTAAGA |
| **B13598** | *RAS1* 3’-screening primer SO2 | TCTACCAGAGAAATATTTTT |
| **B13599** | *RAS1* Southern blot probe primer PO | TCATGTACTGCTCTCTCATG |
| **B13993** | *RAS1* Internal screening primer LP | GAGGGTCAAAGATTCCGACA |
| **B13994** | *RAS1* Internal screening primer RP | TGTCTTGTTGTTGCCACCAT |
| **B13646** | *GPA2* 5’-flanking region primer L1 | GTTTTGTCTACTTTCATCAT |
| **B13647** | *GPA2* 5’-flanking region primer L2 | TTCAGATCCACTAGTTCTAGCTGGGTTTGAGGGAGGGTCT |
| **B13648** | *GPA2* 3’-flanking region primer R1 | TTTCCAGAATTTCACTCTTACCTTGAGAGACAGTGGCATA |
| **B13649** | *GPA2* 3’-flanking region primer R2 | GTTTCCACTGAGAGCTCTTC |
| **B13650** | *GPA2* 5’-screening primer SO | GTCACTTACGTCCCTCATAT |
| **B13651** | *GPA2* 3’-screening primer SO2 | TTAAGATTCAGGATGGGACA |
| **B13652** | *GPA2* Southern blot probe primer PO | CTTTTTTCCAGCCCAGTGAC |
| **B13988** | *GPA2* Internal screening primer LP | AAATGTGTTGGATTGCGTCA |
| **B13989** | *GPA2* Internal screening primer RP | TTTGGAAACGGAAATCGAAG |
| **B13586** | *GPR1* 5’-flanking region primer L1 | ATCAATTAAGGCGAAAAGTA |
| **B13587** | *GPR1* 5’-flanking region primer L2 | TTCAGATCCACTAGTTCTAGTTGTAGTTGGGACGAGACAT |
| **B13588** | *GPR1* 3’-flanking region primer R1 | TTTCCAGAATTTCACTCTTATTTTACGAGACGGTCATTGA |
| **B13589** | *GPR1* 3’-flanking region primer R2 | TCCCGACGTGAGCCAGCCTA |
| **B13590** | *GPR1* 5’-screening primer SO | TTGCTTTATATACTGGATGT |
| **B13591** | *GPR1* 3’-screening primer SO2 | TCTCAAACACTATGATGCCC |
| **B13592** | *GPR1* Southern blot probe primer PO | GGTGACGAAACACGAGCCTT |
| **B13991** | *GPR1* Internal screening primer LP | GACTCTAATGGCACCCCTGA |
| **B13992** | *GPR1* Internal screening primer RP | ACGTCCTCACCAATGCTACC |
| **B15075** | *CDC25* 5’-flanking region primer L1 | CAATTCGATAATGCGGTCCT |
| **B15076** | *CDC25* 5’-flanking region primer L2 | TTCAGATCCACTAGTTCTAGCGCAGCATCTCTCCGTTACT |
| **B15077** | *CDC25* 3’-flanking region primer R1 | TTTCCAGAATTTCACTCTTAAATCAGTGGACCGCAGTTCT |
| **B15078** | *CDC25* 3’-flanking region primer R2 | GGGCATCAATTGACTTTTGG |
| **B15079** | *CDC25* 5’-screening primer SO | CATTCACCGTGATGAACCTG |
| **B15080** | *CDC25* 3’-screening primer SO2 | ATTCGCCGCCTTCTTCTTAT |
| **B15083** | *CDC25* Southern blot probe primer PO | GTTCAAGACCGGCTGAACAT |
| **B15081** | *CDC25* Internal screening primer LP | TCAGCTGGTTGACTTGAACG |
| **B15082** | *CDC25* Internal screening primer RP | AATTGAGGTAAGGCCGTGTG |
| **B15084** | *IRA2* 5’-flanking region primer L1 | ATCCTATCGGCACCTTCTGA |
| **B15085** | *IRA2* 5’-flanking region primer L2 | TTCAGATCCACTAGTTCTAGCAATGTCGAGCCATCTTTGA |
| **B15086** | *IRA2* 3’-flanking region primer R1 | TTTCCAGAATTTCACTCTTAGAACGAGTGCGACATGAAGA |
| **B15087** | *IRA2* 3’-flanking region primer R2 | ATGATCTTGAAGCGGAAACC |

| | | |
|---|---|---|
| **B15088** | *IRA2* 5’-screening primer SO | TCAACCCGGGCAAGTATATC |
| **B15089** | *IRA2* 3’-screening primer SO2 | AAGTAACGTGGGCAGTCACC |
| **B15092** | *IRA2* Southern blot probe primer PO | TGCGATTCATTTGACAATGC |
| **B15090** | *IRA2* Internal screening primer LP | ACGCTTTGCTTGAACGAGAT |
| **B15091** | *IRA2* Internal screening primer RP | GCACGCTGACTCGTGAAATA |
| **B14856** | *PDE1* 5’-flanking region primer L1 | CCAAAAGGTGGAGAACCAGA |
| **B14857** | *PDE1* 5’-flanking region primer L2 | TTCAGATCCACTAGTTCTAGTGGAGGTTGGAAGAAAATGG |
| **B14858** | *PDE1* 3’-flanking region primer R1 | TTTCCAGAATTTCACTCTTATGAGATTAGGGCACCTCTCC |
| **B14859** | *PDE1* 3’-flanking region primer R2 | ATCTCACGCGAAGGTTCTGT |
| **B14860** | *PDE1* 5’-screening primer SO | AATTGGTGCCTTACGACCTG |
| **B14861** | *PDE1* 3’-screening primer SO2 | GCAGCCATTGCTCACAACTA |
| **B14864** | *PDE1* Southern blot probe primer PO | GGGCTTGATCAACAAAGAGC |
| **B14862** | *PDE1* Internal screening primer LP | GCTCAGGCCATTTACTGCTC |
| **B14863** | *PDE1* Internal screening primer RP | ATAAGGTGTTCGGGCATGAG |
| **B14865** | *PDE2* 5’-flanking region primer L1 | AGCCAGAGGACAGCGATAAA |
| **B14866** | *PDE2* 5’-flanking region primer L2 | TTCAGATCCACTAGTTCTAGGGAACGAGGTGAAGAAGGAG |
| **B14867** | *PDE2* 3’-flanking region primer R1 | TTTCCAGAATTTCACTCTTACGCAAATGGGTATCCTATTG |
| **B14868** | *PDE2* 3’-flanking region primer R2 | GGGCAGGAGAGTACAGCAAG |
| **B14869** | *PDE2* 5’-screening primer SO | TGATTCGGCTTCCCTAATTG |
| **B14870** | *PDE2* 3’-screening primer SO2 | CTGCAGCAGACTTTGAGCTG |
| **B14873** | *PDE2* Southern blot probe primer PO | ACGGAATTTGACGGACTGAG |
| **B14871** | *PDE2* Internal screening primer LP | GTGTTTCCTTTGGCACCTGT |
| **B14872** | *PDE2* Internal screening primer RP | TTGAAGCTCGCCTCTCTCTC |
| **B14736** | *RAS1* 5’-flanking region primer L1 (HYG) | AAGAGGCAAATGCGAGACAT |
| **B14570** | *RAS1* 5’-flanking region primer L2 (HYG) | GCACTTTTCGGGGAAATGTGAACTTATACTCTTTTAACAT |
| **B14571** | *RAS1* 3’-flanking region primer R1 (HYG) | CGATGAGGTAGTGCAAGTTAGGTGTTGTGTGATTGTGTAG |
| **B14737** | *RAS1* 3’-flanking region primer R2 (HYG) | AAAAGCTGAACTTGGCAGGA |
| **B13597** | *RAS1* 5’-screening primer SO (HYG) | TGATGGAGATTCGTGTAAGA |
| **B13598** | *RAS1* 3’-screening primer SO2 (HYG) | TCTACCAGAGAAATATTTTT |
| **B13599** | *RAS1* Southern blot probe primer PO (HYG) | TCATGTACTGCTCTCTCATG |
| **B14899** | *RAS1* Internal screening primer LP (HYG) | GGCGGTGGTGTCGGTAAA |
| **B13994** | *RAS1* Internal screening primer RP (HYG) | TGTCTTGTTGTTGCCACCAT |
| **B14856** | *PDE1* 5’-flanking region primer L1 (HYG) | CCAAAAGGTGGAGAACCAGA |
| **B15149** | *PDE1* 5’-flanking region primer L2 (HYG) | GCACTTTTCGGGGAAATGTGTGGAGGTTGGAAGAAAATGG |
| **B15150** | *PDE1* 3’-flanking region primer R1 (HYG) | CGATGAGGTAGTGCAAGTTATGAGATTAGGGCACCTCTCC |
| **B14859** | *PDE1* 3’-flanking region primer R2 (HYG) | ATCTCACGCGAAGGTTCTGT |
| **B14860** | *PDE1* 5’-screening primer SO (HYG) | AATTGGTGCCTTACGACCTG |
| **B14861** | *PDE1* 3’-screening primer SO2 (HYG) | GCAGCCATTGCTCACAACTA |
| **B14864** | *PDE1* Southern blot probe primer PO (HYG) | GGGCTTGATCAACAAAGAGC |
| **B14862** | *PDE1* Internal screening primer LP (HYG) | GCTCAGGCCATTTACTGCTC |
| **B14863** | *PDE1* Internal screening primer RP (HYG) | ATAAGGTGTTCGGGCATGAG |

| | | |
|---|---|---|
| **B17979** | *BCY1* 5’ flanking region primer 1 (*TEF1* promoter replacement) | CTTTCACAAAGTGGTCCAAGG |
| **B17980** | *BCY1* 5’ flanking region primer 2 (*TEF1* promoter replacement) | GTATTCTGGGCCTCCATGTCTGCAAATTAAATTGATATCG |
| **B17981** | *BCY1* 3’ flanking region primer 1 (*TEF1* promoter replacement) | CTACTTCACATCAATTAAACATGGCTCCGTTCGCTACAGA |
| **B17982** | *BCY1* 3’ flanking region primer 2 (*TEF1* promoter replacement) | TCGGCTGAGACAGAGGTTCT |
| **B10742** | *BCY1* Screening primer (*TEF1* promoter replacement) | GGTTTTGCTTCTTCTGCG |
| **B11103** | pV1025 forward-extended primer | CTAGAACTAGTGGATCTGAA |
| **B11104** | pV1025 reverse-extended primer | TAAGAGTGAAATTCTGGAAA |
| **B11105** | NAT spilt primer 1 | CCATTGACTAAGGTTTTCCC |
| **B11106** | NAT spilt primer 2 | TTCAGTAGCCAAACCCATC |
| **B11107** | pV1025 Diagnostic screening primer 1 | TCAGTGGCAAATCCTAACC |
| **B11108** | pV1025 Diagnostic screening primer 2 | AGAGAAAATACCCGTGACG |
| **B12462** | pYM70 forward-extended primer | CACATTTCCCCGAAAAGTGC |
| **B12463** | pYM70 reverse-extended primer | TAACTTGCACTACCTCATCG |
| **B12464** | HYG spilt primer 1 | TGCTGATTTGTCTCAAACTT |
| **B12465** | HYG split primer 2 | TACCATTATCAGTCAAAACA |
| **B12486** | pYM70 Diagnostic screening primer 1 | TGCGGCACAATTGAATAGGG |
| **B12487** | pYM70 Diagnostic screening primer 2 | CGGTGATGACGGTGAAAACC |
| **B16376** | pTO149 forward-extended primer | GACATGGAGGCCCAGAATAC |
| **B16377** | pTO149 reverse-extended primer | CAGTATAGCGACCAGCATTC |
| **B16378** | NEO spilt primer 1 | TGCTCCTGCCGAGAAAGTAT |
| **B16379** | NEO spilt primer 2 | GCTCTTCGTCGAGATCATCC |
| **B16380** | pTO149 Diagnostic screening primer 1 | ACATGGGGATGTATGGGCTA |
| **B16381** | pTO149 Diagnostic screening primer 2 | TTTTCGCCTCGACATCATCT |
| **B16376** | pNEO-TEF1 forward-extended primer | GACATGGAGGCCCAGAATAC |
| **B17918** | pNEO-TEF1 reverse-extended primer | TTTTGTTAGTTTTTGAGGTG |
| **B14393** | CLP for *RAS1* complementation | ACTAGTAAGCGCGCAGGAACGAAACC |
| **B14394** | CRP for *RAS1* complementation | ACTAGTGAGAACGTCAAGACAATTCC |
| **B14397** | Screening primer for *RAS1* complementation | CGTGGAGGTGCAAGTAACGG |
| **B14395** | *RAS1* sequencing primer 1 | TTTGATAGACTAGACAAACG |
| **B14396** | *RAS1* sequencing primer 2 | AGGTGCCGAATTGGCAAAAG |
| **B14483** | CLP for *GPA2* complementation | ACTAGTACTAATGTCTGAGCAGAGTT |
| **B14484** | CRP for *GPA2* complementation | ACTAGTGTCAATGAGCCAAGGACAGA |
| **B14488** | Screening primer for *GPA2* complementation | TGGCAATTTTTCAAGGAACA |
| **B14485** | *GPA2* sequencing primer 1 | CTACGTGAGATTCAGTATGA |
| **B14486** | *GPA2* sequencing primer 2 | TATTCATTTCTTTTTCTTTT |
| **B14487** | *GPA2* sequencing primer 3 | TGGTGAATGCAATCATTGAT |
| **B14801** | CLP for *GPR1* complementation | GCATGCTGCAAGCACATTCCGCACTG |
| **B14802** | CRP for *GPR1* complementation | GCATGCGATGATATGAAGATGCAAGA |
| **B14806** | Screening primer for *GPR1* complementation | GGTCTGCGAGGTTCTCTTTG |
| **B14803** | *GPR1* sequencing primer 1 | CCCAGTGCCAGGCTACTTTT |

| | | |
|---|---|---|
| **B14804** | *GPR1* sequencing primer 2 | TAACCTTTCAGTCAAGGTGC |
| **B14805** | *GPR1* sequencing primer 3 | GCTTTCATGCAACCATTTAA |
| **B15155** | CLP for *CDC25* complementation | CGTTGACACCAACAACGAAC |
| **B15156** | CRP for *CDC25* complementation | GGGCCCTCTGTTCATCTCCGCCTTCT |
| **B15207** | CLP2 for *CDC25* complementation | TTAATTAAGTTATTTATTCG |
| **B15208** | CRP2 for *CDC25* complementation | TTAATTAAGCCATTCATATT |
| **B15164** | Screening primer for *CDC25* complementation | GGTCATCAAACCCTCCTCAA |
| **B15157** | *CDC25* sequencing primer 1 | CTCATTTGCAACCGTTTCGG |
| **B15158** | *CDC25* sequencing primer 2 | CATTCTAATTCTCTCACATG |
| **B15159** | *CDC25* sequencing primer 3 | TTAGTAACGGAGAGATGCTG |
| **B15160** | *CDC25* sequencing primer 4 | CAATGTCAACCAGCAGTTCA |
| **B15161** | *CDC25* sequencing primer 5 | TCCTAGCAGATGAGTTCAAT |
| **B15162** | *CDC25* sequencing primer 6 | TTGTTGAAGAGAGAGAGACA |
| **B15163** | *CDC25* sequencing primer 7 | CAGATTAATAAGCTTGCCTG |
| **B16075** | CLP for *IRA2* complementation | TTTACAACATATAAGAAACT |
| **B16076** | CRP for *IRA2* complementation | GGGCCCACTTCGAAACCATAAAGGTG |
| **B16077** | CLP2 for *IRA2* complementation | TCTAGAGAAAGCAATGTTCA |
| **B16078** | CRP2 for *IRA2* complementation | TCTAGAGTTCCGCAACATGG |
| **B15175** | Screening primer for *IRA2* complementation | GAAGCCCGTGAACGACGCCG |
| **B15167** | *IRA2* sequencing primer 1 | CTTCATTTTCTTGAACAAGT |
| **B15168** | *IRA2* sequencing primer 2 | ACTGCAATCAAAAATGATGG |
| **B15169** | *IRA2* sequencing primer 3 | AAACTAGTGCATGAAATGGC |
| **B15170** | *IRA2* sequencing primer 4 | CCCGTTCCATAGCTTAACGG |
| **B15171** | *IRA2* sequencing primer 5 | TGTAATCTACAACGACTGCC |
| **B15172** | *IRA2* sequencing primer 6 | CTAGGCAATTTTGTCGCATT |
| **B15173** | *IRA2* sequencing primer 7 | GCGATCACTCTTGTCGGTAC |
| **B15174** | *IRA2* sequencing primer 8 | CCTTCGGTTGTACAACCATC |
| **B15069** | CLP for *PDE1* complementation | AGAAGGTGACCAAAGAAGAA |
| **B15070** | CRP for *PDE1* complementation | CTTATTACCATCAATGAGAA |
| **B15074** | Screening primer for *PDE1* complementation | TCAATTGCGATTCCTTCCCA |
| **B15071** | *PDE1* sequencing primer 1 | AATAAGTGCAGATCTTCGGT |
| **B15072** | *PDE1* sequencing primer 2 | ATTGCATCCCCACAATTCTC |
| **B15073** | *PDE1* sequencing primer 3 | GAAGCAACCTACAAGTCAGC |
| **B15241** | CLP for *PDE2* complementation | ATTCAGGTGGATTTCTTGAT |
| **B15242** | CRP for *PDE2* complementation | CCACTGGAGGCAGCGAAAGC |
| **B15247** | Screening primer for *PDE2* complementation | CGGTGTTTGACATGCTCAAG |
| **B15243** | *PDE2* sequencing primer 1 | AGAGTAAATTTAAAGACAGC |
| **B15244** | *PDE2* sequencing primer 2 | CACCGCACCACAGTTGATTA |
| **B15245** | *PDE2* sequencing primer 3 | AAATCTACAATGAAAGATCG |
| **B15246** | *PDE2* sequencing primer 4 | TAGATTTGCTCGTAGTAGTG |
| **B11749** | *ACT1* qRT-PCR primer 1 | TTGCTCCTGAAGAACACCCT |

| | | |
|---|---|---|
| **B11750** | *ACT1* qRT-PCR primer 2 | GCAGGAACGTTGAAGGTCTC |
| **B16935** | *RAS1* qRT-PCR primer 1 | ATGGTGGCAACAACAAGACA |
| **B16936** | *RAS1* qRT-PCR primer 2 | AACACCCGCTCTTCTCCTTT |
| **B16937** | *CDC25* qRT-PCR primer 1 | ACTGGTGCAACCCATTCTTC |
| **B16938** | *CDC25* qRT-PCR primer 2 | CAGCGCTGTCAATGATGACT |
| **B16941** | *PDE1* qRT-PCR primer 1 | GGTGGAGTTGGGTGCTCTAA |
| **B16942** | *PDE1* qRT-PCR primer 2 | TATTGCCATGCTGAAGGTCA |
| **B16939** | *PDE2* qRT-PCR primer 1 | CGATCCCAGAGAGAATGAGC |
| **B16940** | *PDE2* qRT-PCR primer 2 | ACAGGTGCCAAAGGAAACAC |
| **B17723** | *HSP21* qRT-PCR primer 1 | GACGTTGGACCTGAAGGTGT |
| **B17724** | *HSP21* qRT-PCR primer 2 | GTCGCCTTCAATCTTCTTGG |
| **B17725** | *HSP60* qRT-PCR primer 1 | GTGAGGAGGGTGCTGTCATT |
| **B17726** | *HSP60* qRT-PCR primer 2 | CAGCAATCATGTCGGTGAAC |
| **B14119** | *HSP70* qRT-PCR primer 1 | TGGGAACCACGTATTCCTGT |
| **B14120** | *HSP70* qRT-PCR primer 2 | AGGCAACGTATGAGGGTGTC |
| **B14121** | *HSP78* qRT-PCR primer 1 | GAACCGTTTGGACGAAGTGT |
| **B14122** | *HSP78* qRT-PCR primer 2 | TCGCCAATCTCTCTCAACCT |
| **B12994** | *HSP90* qRT-PCR primer 1 | TTCCGCTGGTGCCGATGTCT |
| **B12995** | *HSP90* qRT-PCR primer 2 | GGACGTGGTCGGCAACCAAGAA |
| **B14123** | *HSP104* qRT-PCR primer 1 | GGATGGTGCCTTTGAAAGAA |
| **B14124** | *HSP104* qRT-PCR primer 2 | AGGCTGCAAACCTCTCAAAA |
| **B16784** | *ALS4* qRT-PCR primer 1 | GGTGCTATCACGGACCAAGT |
| **B16785** | *ALS4* qRT-PCR primer 2 | CAGTGGCTTGATACGACGAA |
| **B16786** | *PGA7* qRT-PCR primer 1 | ATTATGGCCACAGCGAAAAC |
| **B16787** | *PGA7* qRT-PCR primer 2 | CAACAAAACCACCCCAAAAC |
| **B16788** | *SIT1* qRT-PCR primer 1 | GGTGCCGCTACTGGTGTATT |
| **B16789** | *SIT1* qRT-PCR primer 2 | GGAAGCTGACGCTACCACTC |
| **B17623** | *SAP6* qRT-PCR primer 1 | TTTGGCATGTATCGCTTGAG |
| **B17624** | *SAP6* qRT-PCR primer 2 | TCAGAGAGAGGCACCCTGAT |
| **B17633** | *PGA26* qRT-PCR primer 1 | GGCTACTCTAACGCCACCAC |
| **B17634** | *PGA26* qRT-PCR primer 2 | TGCAAACCTCCTCACACTTG |
| **B11755** | *BCY1* qRT-PCR primer 1 | AATGGCACCAAGGTCAACAC |
| **B11756** | *BCY1* qRT-PCR primer 2 | ATGCCCAACAAGTCACATCG |

## References

1. Munoz JF, Gade L, Chow NA, Loparev VN, Juieng P, Berkow EL, Farrer RA, Litvintseva AP, Cuomo CA. 2018. Genomic insights into multidrug-resistance, mating and virulence in *Candida auris* and related emerging species. Nat Commun **9**:5346.

2. Kim JS, Lee KT, Lee MH, Cheong E, Bahn YS. 2021. Adenylyl cyclase and protein kinase A play redundant and distinct roles in growth, differentiation, antifungal drug resistance, and pathogenicity of *Candida auris*. mBio **12**:e0272921.
